# Comprehensive profiling of volatile aroma compounds and non-volatile metabolites in five salted chili peppers using multi-omics technologies: *E*-nose, *E*-tongue, GC-IMS, GC × GC-TOF-MS, and UHPLC-MS/MS

**DOI:** 10.1016/j.fochx.2026.103714

**Published:** 2026-03-04

**Authors:** Wen-ke Yang, Rui Deng, Ya-qin Huang, Hui-ping Xia, Ke-yao Wang, Yi Zhou, Ming-xi Liao, Qing-ming Li

**Affiliations:** a College of Food Science and Technology, Hunan Agricultural University, Changsha 410128, Hunan, China; b Engineering Research Center for Pepper Deep Processing of Hunan Province, Changsha 410128, Hunan, China; c Lameizi Foodstuff Co., Ltd., Yiyang 413100, Hunan, China

**Keywords:** Salted chili pepper, Volatile compounds, Non-volatile metabolites, GC × GC-TOF-MS, UHPLC-MS/MS, Correlation analysis

## Abstract

This study aimed to decipher the flavor profiles of salted chili peppers (SCP) from five geographical origins and elucidate the molecular basis of their characteristic savory-aromatic fusion. Utilizing a multi-omics approach on samples of the same variety processed identically, we identified 75 characteristic odor-active compounds. SCP from Guizhou (SCP3) exhibited the most intense aroma, rich in esters and phenols. Umami attributes were primarily driven by amino acids, peptides, and lipids, with Shandong SCP4 showing the highest umami potential. Critically, correlation analysis revealed significant synergistic effects between key aroma compounds and umami metabolites. The findings demonstrate that geographical origin is the primary driver of flavor variation in SCP. The uncovered synergy provides molecular-level insight into the “savory-aromatic” harmony, establishing a foundation for quality control and flavor enhancement.

## Highlights

• The umami characteristics of salted chili peppers (SCP) was prominent.
• 75 characteristic OACs were identified in five distinct geographical SCPs.
• The Guizhou SCP featured a high concentration of esters and phenols.
• Non-targeted metabolomics reveals 49 differential metabolites in five SCPs.

## 1 Introduction

Hunan chili sauce is one of the traditional fermented chili products in China. Distinct from similar sauces found in other regions, Hunan chili sauce not only exhibits a notable spiciness but also showcases a prominent umami flavor along with unique and diverse fragrance characteristics. It imparts the distinctive umami and aroma that are central to the flavor profile of Hunan cuisine (Xu et al., 2025). Currently, the common industrial production method for Hunan chili sauce primarily involves a two-stage process: fresh chili peppers are first preserved through salting to produce salted chili peppers (SCP), which are then desalted and ground to yield the final sauce product. Consequently, as a critical intermediate, the flavor profile of SCP directly determines the quality of the final chili sauce. Typically, the SCP used by manufacturing facilities in Hunan is sourced from diverse geographical regions such as Shandong and Hebei. At these origins, fresh chilies are uniformly processed into SCP through identical, standardized procedures for washing, grading, and salting, before the SCP is transported to Hunan for final sauce production.

Previous studies have identified characteristic flavor compounds in pickled chili peppers and chopped pepper. Chopped pepper exhibits a complex overall flavor perception characterized by aromatic notes of fruit, wood, and peppery spiciness, resembling chili sauce, while its non-volatile metabolites are rich in proteins, dietary fiber, essential amino acids, unsaturated fatty acids, and mineral (Chen et al., 2024). The overall flavor perception of pickled chili peppers is a composite of pungency, aroma, and taste. Specifically, the primary volatile organic compounds (VOCs) in pickled chili peppers comprise esters, aldehydes, alcohols, ketones, sulfur-containing compounds, and terpenes (Tang et al., 2024). Furthermore, non-volatile metabolites in pickled chili peppers primarily comprise organic acids, free amino acids, capsaicinoids, and inorganic salts (Tang et al., 2024).

A study analyzing 27 traditional spontaneously fermented sauerkraut samples from nine different cities in Heilongjiang Province, Northeast China, found significant regional variations in their flavor profiles (Wang et al., 2023). Similarly, a study comparing traditionally pickled mustard tuber from Ningbo, Inner Mongolia, and Harbin in China revealed significant regional differences in their physicochemical properties and bacterial communities, which directly influence their distinct taste and flavor profiles (Liu et al., 2024). Therefore, regional differences are recognized as a key factor shaping the diverse flavor profiles of traditional fermented vegetables.

However, existing research has predominantly centered on chopped and pickled chili peppers, leaving the flavor profile of SCP inadequately characterized. Crucially, the relationship between the characteristic aroma compounds and non-volatile umami metabolites that define SCP flavor remains elusive, and the molecular basis for their potential synergy has yet to be elucidated. Moreover, an integrated analytical approach employing multiple precision instruments for comprehensive SCP assessment has not been documented.

Accordingly, this study aims to characterize the overall flavor and umami properties of five geographically distinct SCPs and to elucidate the relationship between them. To this end, the overall flavor was objectively described using electronic nose (*E*-nose) and electronic tongue (E-tongue). Characteristic volatile and non-volatile metabolites were then identified by GC-IMS, GC × GC-TOF-MS, and UHPLC-MS/MS, respectively, with the complementarity between the two gas chromatography platforms also evaluated. Finally, the interaction between aroma and taste compounds was revealed through cross-omics correlation analysis. This work provides a comprehensive perspective for assessing the flavor quality of SCP.

## 2 Materials and methods

### 2.1 Sample preparation

The SCP samples were sourced from the raw material supply bases of Lameizi Foodstuff Co., Ltd. Fresh Chinese Horn pepper (*Capsicum annuum* L.) of the same variety, harvested at the commercial red-ripe stage in September 2024, were collected from five representative producing regions. These peppers were selected for uniform size and absence of physical defects, and were processed using an identical traditional salting procedure to prepare SCP. Following harvest, the peppers were promptly transported under refrigerated conditions (4 °C) to the centralized processing facility, where the salting procedure was completed within 24 h of arrival. The standardized salting process was carried out in dedicated pepper salting tanks (measuring 5.55 m in length, 2.75 m in width, and 2.95 m in height), using a balanced brine concentration of 20%, with a total salting duration of two months. After processing, the SCP samples were coded according to their geographical origins as follows: SCP1 (Jiangsu), SCP2 (Hebei), SCP3 (Guizhou), SCP4 (Shandong), and SCP5 (Henan). The samples were then transported to the laboratory and stored at −80 °C until analysis. To ensure sample homogeneity, each sample was thoroughly homogenized using a commercial blender prior to instrumental analysis.

### 2.2 *E*-nose analysis

A PEN3 E-nose (Airsense Analytik GmbH, Germany) was employed according to the method described by Ju et al. (Ju et al., 2025) with minor modifications. Sample preparation involved weighing 2.0 g of each SCP into a 20 mL headspace vial, followed by equilibration at 25 °C for 40 min. Measurement conditions were set as follows: injection flow rate 200 mL/min, acquisition time 120 s, and chamber purging with clean air between samples to ensure baseline recovery.

### 2.3 *E*-tongue analysis

Weigh 10 g of the homogenized sample separately, dissolve it in 100 mL of purified water at 40 °C, and stir thoroughly with shaking. Allow the mixture to stand for 30 min, then filter the supernatant through neutral filter paper. Finally, transfer 40 mL of the filtrate into a sample cup for E-tongue testing. Measurement time: 120 s; stirring speed: 1 r/s (Zhang et al., 2021).

### 2.4 GC-IMS analysis

VOCs were analyzed using a FlavourSpec® flavor analyzer (G.A.S. GmbH, Germany). Homogenized samples (1.0 g) were incubated in 20 mL headspace vials at 40 °C for 30 min with 500 rpm shaking. Then, 500 μL of headspace gas was injected at 85 °C into an MXT-WAX column (30 m × 0.53 mm, 1 μm film) held at 60 °C, using nitrogen as carrier and drift gas (Li et al., 2022). The IMS temperature was 45 °C, and total run time was 30 min. Compounds were identified using the built-in NIST 2020 GC retention index and IMS migration time databases in VOCal software. Visualization and comparative analysis were performed using Reporter, Gallery Plot, and Dynamic PCA plugins.

### 2.5 GC × GC-TOF-MS analysis

VOCs were analyzed using an Agilent 8890 GC coupled with an Agilent 7250 Q-TOF mass spectrometer. The analysis method was referred to the previous literature with some modifications (Zhao et al., 2025). Samples (1.0 g) were placed in 20 mL headspace vials with 1.5 mL saturated NaCl solution and 20 μL 2-octanol (0.16 mg/mL) as internal standard. The vial was sealed and equilibrated at 70 °C for 10 min. The SPME fiber type (50/30 μm DVB/CAR/PDMS), extraction temperature (70 °C), and extraction time (10 min) were optimized based on preliminary single-factor experiments to maximize the extraction efficiency of target volatile compounds in SCP samples. Volatiles were extracted using SPME fiber at 70 °C for 10 min, then desorbed at 240 °C for 5 min.

Separation used an Agilent DB-Wax column (60 m) with hydrogen carrier gas at 1 mL/min. The temperature program was: 40 °C (hold 2 min), raised to 80 °C at 4 °C/min (hold 1 min), then to 240 °C at 3 °C/min (hold 4 min). MS conditions were: EI mode at 70 eV, quadrupole 150 °C, ion source 200 °C, transfer line 280 °C, mass range 45–350 amu. Raw data underwent peak identification, mass deconvolution, and retention index calculation (based on the C6-C27 n-alkane series) using Canvas software. VOCs identification required simultaneous fulfillment of the following criteria: mass spectrum match factor ≥ 700, reverse match factor ≥ 800, and retention index deviation from NIST database reference values within ±15.

### 2.6 Non-volatile metabolite analysis

#### 2.6.1 Metabolite extraction

After slowly thawing the sample at 4 °C, add an appropriate amount to pre-chilled methanol/acetonitrile/water solution (2:2:1, *v*/*v*/v). Vortex mix, sonicate at low temperature for 30 min, then let stand at −20 °C for 10 min. Centrifuge at 14,000 *g* at 4 °C for 20 min. Remove the supernatant and vacuum-dry it. For mass spectrometry analysis, resuspend the dried residue in 100 μL of acetonitrile/water solution (acetonitrile: water = 1:1, v/v), vortex, centrifuge at 14,000 *g* at 4 °C for 15 min, and inject the supernatant for analysis.

#### 2.6.2 UHPLC-MS/MS analysis

Non-Volatile metabolites in SCP were determined by UHPLC-MS/MS. Chromatographic separation was done using an Agilent 1290 Infinity UHPLC system equipped with a ACQUITY UPLC BEH Amide column (1.7 μm, 2.1 × 100 mm) maintained at 25 °C. The mobile phase consisted of (A) 25 mM ammonium acetate/ammonia in water and (B) acetonitrile. The gradient elution program was: 0–0.5 min at 95% B; 0.5–7 min from 95% to 65% B; 7–8 min from 65% to 40% B; 8–9 min at 40% B; 9–9.1 min from 40% to 95% B; and 9.1–12 min at 95% B. The flow rate was set to 0.5 mL/min with a 2 μL injection volume. To reduce signal fluctuations from the instrument, samples were randomized using a computer-generated random number list. Quality control (QC) samples were prepared by pooling equal aliquots of all experimental samples. Three QC injections were strategically placed: one at the beginning (after system equilibration), one in the middle, and one at the end of the analytical sequence to monitor system stability and data reliability. Mass spectrometric analysis was performed on an AB Triple TOF 6600 system using both positive and negative electrospray ionization modes. Data were collected in data-dependent acquisition (DDA) mode. Full-scan MS^1^ spectra (*m*/*z* 60–1000) were acquired with a 0.20 s accumulation time. The ten most intense precursor ions from each scan were selected for fragmentation at 35 ± 15 eV collision energy. The resulting MS^2^ spectra (m/z 25–1000) were collected with a 0.05 s accumulation time. Ion source parameters were: GS1/GS2 at 60 psi, curtain gas at 30 psi, temperature at 600 °C, ion spray voltage at ±5500 V, and declustering potential at ±60 V. Analytical precision was validated using the pooled QC samples, with an acceptance criterion of RSD < 30% for feature reproducibility. High-confidence metabolite identification was achieved by matching against authentic chemical standards, requiring agreement in retention time, mass accuracy (<10 ppm), and MS/MS spectra (MSI confidence level ≧ 2) (Cong et al., 2024). Convert the raw data acquired by mass spectrometry using Proteo Wizard (version 3.0.6150) to generate. Mzxml format files. Then, XCMS (version 1.46.0) was used for peak alignment, retention time correction, and peak area extraction. Import the MGF files into metDNA (http://metdna.zhulab.cn/) for metabolite identification.

#### 2.6.3 Sensory evaluation

A trained panel (*n* = 7) evaluated taste attributes using a 0–9 intensity scale. Reference solutions were prepared for bitterness (caffeine, 0.1 mg/mL threshold), astringency (tannic acid, 0.1 mg/mL), umami (MSG, 0.8 mg/mL), and saltiness (NaCl, 2.0 mg/mL). Four concentrations (1×, 2×, 4×, 6× threshold) were anchored to 1, 3, 6, and 9 points, respectively. Aftertaste (defined as lingering flavor intensity) was referenced to a 3.0 mg/mL MSG solution (6 points). Panelists were trained to internalize these scales before evaluating samples in triplicate under blind conditions (Zhao et al., 2023).

### 2.7 Data analysis

In triplicate, samples were examined for non-volatile metabolites and fragrance chemicals. The Meggie Bio Cloud platform (https://cloud.majorbio.com) was used to visualize metabolite data, and Origin software (Version 2021, Origin Lab, Northampton, MA, USA) was used to create heat maps. SIMCA 14.1 (Umetrics, Umea, Sweden) was used for principal component analysis (PCA) and orthogonal partial least squares-discriminant analysis (OPLS-DA). SPSS 27.0.1 (IBM, Armonk, USA) was used to analyze GC-IMS data. Using Origin software, connections between non-volatile metabolites and fragrance molecules were investigated using Pearson's correlation analysis.

## 3 Results and discussion

### 3.1 Flavor profile characterization by *E*-nose and E-tongue

**Fig. 1:**
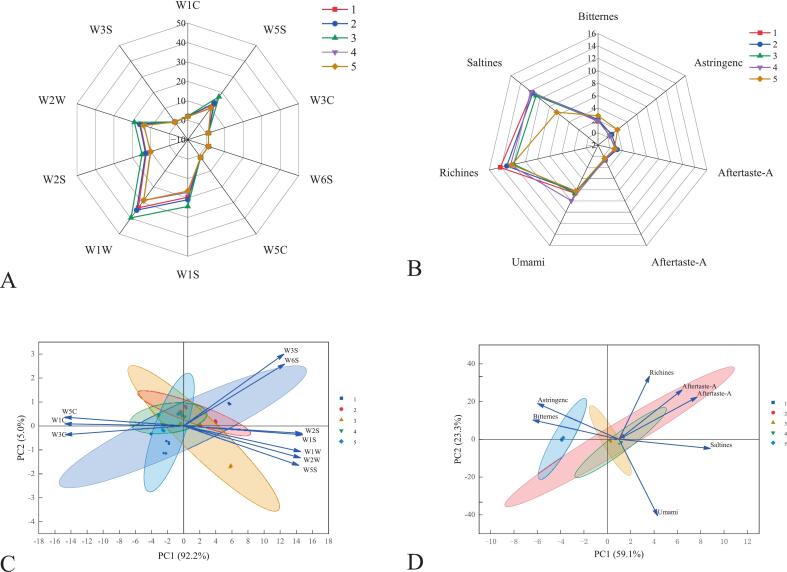
Radar chart (A) and principal component analysis (C) of *E*-nose data for five SCPs. Radar chart (B) and principal component analysis (D) of E-tongue data for five SCPs. Sample codes in the fig. (1–5) represent SCP1 to SCP5, respectively.

The radar map generated from the E-nose analysis provided a clear visualization of the distinct VOC profiles among the five SCPs from different regions (Fig. 1A). All samples exhibited strong responses to sensors W1W (sensitive to sulfides and terpenes), W1S (sensitive to short chain alkanes), W2W (sensitive to organic sulfides and aromatic components), and W5S (sensitive to nitrogen oxides). This observation indicated that the volatile characteristics captured by these sensors are consistently significant across all samples. They collectively form the characteristic chemical basis underlying both the similarities and differences in flavor profiles among all sample types. Notably, SCP3 displayed a substantially larger enclosed area, indicating a significantly greater overall intensity of VOCs and suggesting a more complex aromatic profile compared to the other samples. Meanwhile, distinct clustering patterns were revealed in the chart. Specifically, SCP1 and SCP2 converged on sensors W3C (sensitive to aromatic ammonia), W6S (mainly selective to hydrogen), and W5S, but diverged markedly on sensors W1W and W1S. Furthermore, the radar plots of SCP4 and SCP5, which showed the smallest enclosed area and a high degree of overlap, implied a remarkably similar volatile composition.

PCA (Fig. 1C) effectively distinguished the samples, with PC1 and PC2 accounting for 97.2% of total variance. SCP 1, SCP2 and SCP3 clustered in the positive region of PC1, correlating with sulfide and alcohol/aldehyde/ketone sensors. In contrast, SCP 4 and SCP5 scattered in the negative region of PC1, which was associated with aromatic and olefin compounds. These results demonstrated fundamental flavor differences among the five SCPs, primarily driven by varying contents of sulfur compounds and nitrogen oxides. This provided a foundation for subsequent chromatographic analysis of characteristic differential volatile.

The *E*-tongue measurement used artificial saliva as the reference standard. The analytical results (Fig. 1B) indicated that umami was a prominent characteristic of SCP, exhibiting significant variations in umami response values among different samples. These variations might be attributed to differences in the content of substances such as free amino acids during the fermentation process (Zhao et al., 2016). The PCA of the *E*-tongue data (Fig. 1D) revealed clear taste distinctions among the samples. PC1 (59.1%) and PC2 (23.3%) collectively accounted for 82.4% of cumulative variance, which reflected the overall sample characteristics. The clustering pattern observed for SCP5 along the negative axis of PC1 alongside other taste sensors could be due to its relatively high levels of astringent and bitter constituents. Conversely, SCP1, SCP2, SCP3, and SCP4 clustered along the positive PC1 axis associated with umami and saltiness, likely due to their similar responses to salty and umami tastes. The E-tongue results revealed distinct umami intensity and taste differentiation patterns among SCPs (Fig. 1). To decipher the specific non-volatile metabolites responsible for these sensory patterns, we subsequently performed a comprehensive non-targeted metabolomic analysis using UHPLC-MS/MS.

### 3.2 Comprehensive analysis of VOCs

#### 3.2.1 Rapid fingerprint analysis and preliminary identification of VOCs using GC-IMS

**Fig. 2:**
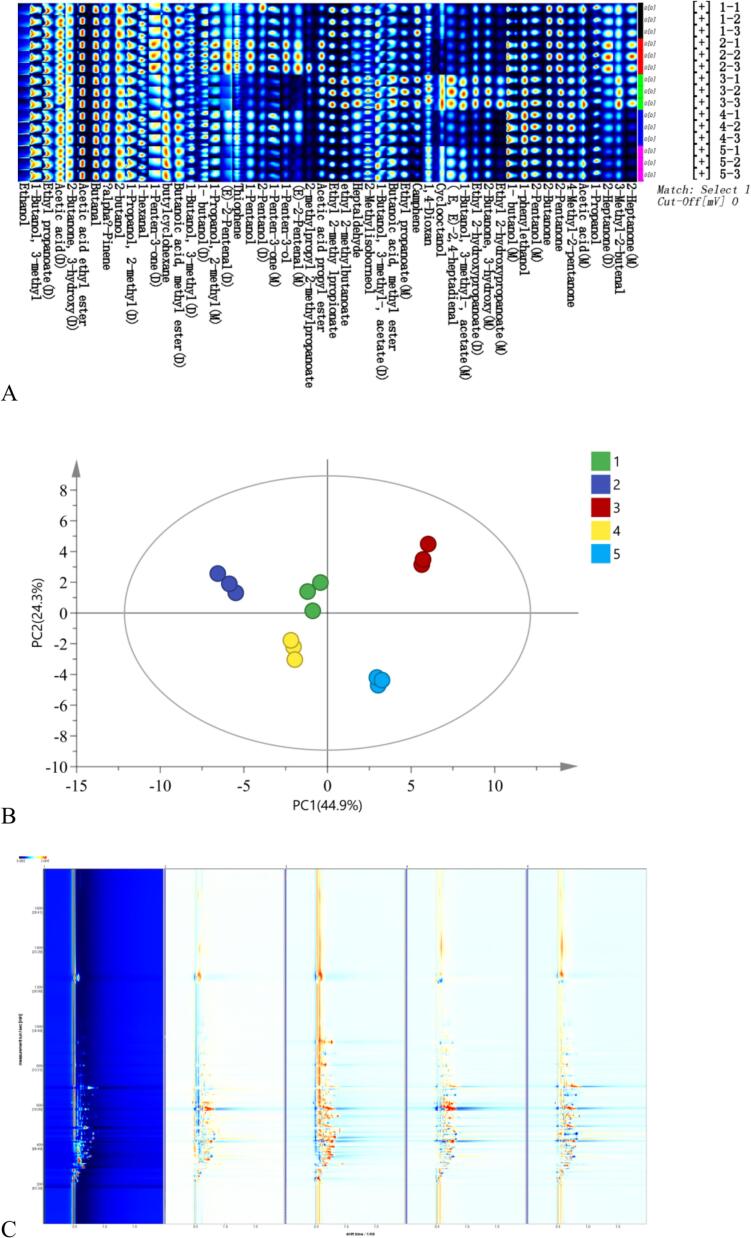
Spectra of five SCPs from different origins obtained via GC-IMS. Sample codes in the fig. (1–5) represent SCP1 to SCP5, respectively. (A) Dynamic fingerprint spectra of SCP from five different origins generated via Gallery Plot. Each row represents signal peaks from a single sample, while each column corresponds to the same volatile compounds across different samples. Color intensity indicates VOC concentration, with brighter hues denoting higher levels. (B) PCA score plot of complete signals. (C) Dynamic differential spectrum.

GC-IMS fingerprinting provided a rapid visual overview, confirming significant qualitative and quantitative differences in VOC profiles among the SCPs. The spectral comparison diagram (Fig. 2A) provided a direct visual comparison, where each point represented a specific VOC. Its intensity correlated with concentration, making qualitative and quantitative differences in VOC spectra immediately apparent. Through qualitative analysis utilizing GC-IMS database, multiple characteristic flavor compounds were preliminarily identified, including aldehydes (e.g., hexanal, 3-methylbutanal), ketones (e.g., 2-heptanone, 2-butanone), alcohols (e.g., ethanol, 1-pentanol), and esters (e.g., ethyl acetate, 3-methylbutyl acetate). Notably, within the regions corresponding to aldehydes and ketones (e.g., within the retention time range of approximately 400–800 s and migration rate of 1.0–1.3), the signal intensities of SCP3 and SCP5 (manifested as distinct red spots) were significantly stronger than those observed for SCP1 and SCP4. This observation suggested that SCP3 and SCP5 may contain higher concentrations of characteristic volatile compounds responsible for imparting grassy, fruity, and fatty notes. Furthermore, in the ester distribution region, SCP2 also exhibited a relatively strong signal intensity indicative of more pronounced fruity notes. Overall, this fingerprint spectrum visually revealed significant differences in the aroma profiles among the five SCPs while establishing a foundation for subsequent statistical analysis.

PCA of the GC-IMS data quantified the observed differences, revealing clear geographical clustering patterns. The PCA was performed based on the peak areas of all detected VOCs across samples. Prior to PCA, the data were normalized to total peak area to account for variations in sample concentration and instrument response. The first two principal components (PC1 and PC2) in the PCA score plot (Fig. 2B) captured 69.2% of the cumulative variance, and effectively reflected the majority of variation among the samples. Five SCPs exhibited clear separation and clustering trends in PCA space. SCP1 and SCP2 clustered closely in the region where both PC1 and PC2 have negative values, indicating that their overall volatile flavor profiles are highly similar. Conversely, SCP3 was isolated in the region where both PC1 and PC2 were located the positive semi-axis. SCP4 resided in the region where PC1 was on the negative semi-axis and PC2 was on the negative semi-axis. In contrast, SCP5 occupied a region where PC1 was on the positive semi-axis and PC2 was on the negative semi-axis. Both samples maintained a considerable distance from the others, indicating their distinct flavor characteristics. The PCA results provided strong objective statistical confirmation that the overall VOC profiles of the five SCPs exhibited significant differences. This finding corroborated the intuitive observations from the fingerprint spectra (Fig. 2A) and quantified the complex relationships among samples into a clear spatial distribution model.

To visualize differences, a differential comparison was performed by subtracting the spectrum of SCP1 (reference) from those of the other samples. SCP1 was selected as the reference sample because preliminary analysis indicated it exhibited intermediate VOC levels across major compound classes, making it a suitable baseline for comparative analysis. This resulted in the GC-IMS difference images presented in Fig. 2C. If VOCs in both samples are identical, the background after subtraction appears white. The red signals indicate that the concentration of the substance is higher than that in the reference, while the blue signals indicate a lower concentration relative to the reference. The difference map clearly showed that all other samples exhibited extensive and significant differences in their VOC composition when compared to SCP1. Notably, SCP3 displayed a large region with high-intensity continuous red signal across the entire retention time range, particularly between 200 and 1000 s. VOC levels in SCP3, across all major classes (aldehydes, ketones, esters), far exceeded those in the reference (SCP1). Consequently, it can be inferred that SCP3 possesses the most intense and complex overall aroma profile. In contrast, SCP4 and SCP5 also exhibited numerous red signals, these were less intense and more concentrated during later retention period (500–1000 s), which was typically associated with higher molecular weight compounds (Wang et al., 2022). This pattern implied a distinct, albeit still rich, flavor character differentiated from that of SCP3. SCP2 exhibited the weakest differential signal among all samples tested, indicating its VOC profile is most similar to that of the reference (SCP1). These results clearly demonstrated that SCP3 has the most pronounced VOC enrichment, likely forming the core material basis for its unique flavor. It aligns with and provides molecular-level validation for the *E*-nose results, where SCP3 exhibited the largest radar chart area, signaling the greatest overall VOC intensity. Three-dimensional spectrum maps (Fig. S1A) and “nearest neighbor” fingerprint analysis diagrams (Fig. S1B) also demonstrated significant flavor differences among the five samples.

Due to the high resolution capability of GC-IMS for ionization modes, two distinct signals were separated for each compound (Li et al., 2019). Some compounds exhibited both monomers and dimers that displayed similar retention times but different in drift times. For instance, the retention time of 2-Heptanone monomer is 655.14, while that of its dimer is 653.81, with corresponding drift times of 1.63and 1.26 respectively. GC-IMS analysis detected 52 valid signal peaks (Table S1), identifying a total of 40 VOCs. Among these compounds: Alcohols accounted for the largest number with 11 compounds; Esters comprised 9 compounds; Aldehydes comprised 7 compounds; Ketones comprised 6 compounds; Alkenes and heterocyclic compounds each comprised 2 compounds; Acids comprised the smallest number with only 1 compound. Among these, alcohols and esters constituted the primary flavor categories identified by GC-IMS, accounting for 48.7% of all identified compounds. Aldehydes represented 17.9% and were a significant contributor to volatile flavors. Although only one sulfur-containing compound was identified, thiophene a characteristic flavor compound significantly influences the overall flavor profile of SCP.

#### 3.2.2 Screening of significant differential odor-active compounds in GC-IMS based on ROAV and PLS-DA

Characteristic VOCs contributing to sample differentiation were identified through partial least squares discriminant analysis (PLS-DA) combined with variable importance in projection (VIP) of the 40 detected compounds. Furthermore, to assess the sensory significance of these substances, we calculated their relative odor activity values (ROAV). Significant differential odor-active components (OACs) were defined as those compounds which exhibited VIP > 1 or ROAV >1. Ultimately, 18 compounds were identified as differential OACs. The analysis results indicated that these distinct OACs comprise 4 esters, 6 alcohols, 7 aldehydes and ketones, and 1 terpene (Table 1). Among them, 8 compounds with ROAV ≥1 were designated as key OACs, exerting the most direct and significant impact on the sensory profile. The most prominent of these was Ethyl 2-methylpropionate, which exhibited the highest absolute ROAV across all samples, suggesting it may be a major contributor to the product's characteristic aroma, imparting dominant fruity, apple, and strawberry notes. Another ester, ethyl 2-methylbutanoate, along with Butanal and 1-Penten-3-one, also served as key OACs, contributing essential fruity, green, and pungent nuances (Chen et al., 2023). The remaining 10 compounds with 0.1 ≤ ROAV <1, were classified as supplementary OACs. While these substances do not dominate the aroma singularly, they play a crucial role in modulating and enriching the primary fragrance, adding layers of complexity.

**Table 1:** Screening of Characteristic OACs in GC-IMS Based on ROAV and PLS-DA.

Compound Name	VIP			ROAV			Odor description
		SCP1	SCP2	SCP3	SCP4	SCP5	
1-Butanol, 3-methyl-, acetate	1.13	1.42 ± 0.09^c^	1.81 ± 0.21^ab^	2.15 ± 0.34^a^	1.89 ± 0.07^ab^	1.84 ± 0.56^ab^	Banana, Fruity
1-Penten-3-one(D)	1.13	2.43 ± 1.17^ab^	3.67 ± 0.62^c^	1.14 ± 1.54^a^	2.95 ± 0.97^ab^	1.34 ± 1.02^bc^	Pungent, Fishy, Green
1-hexanal	1.10	1.42 ± 0.72^a^	1.78 ± 0.77^a^	0.89 ± 0.84^b^	1.49 ± 1.85^a^	1.00 ± 0.33^ab^	Green, Grassy, Fatty
Ethyl 2-methy lpropionate	1.04	67.76 ± 0.28^b^	61.49 ± 0.33^c^	78.44 ± 0.39^a^	52.59 ± 1.64^d^	39.58 ± 0.55^e^	Fruity, Apple, Strawberry
Butanoic acid, methyl ester	1.72	0.11 ± 0.10^a^	0.14 ± 0.03^a^	0.11 ± 0.08^a^	0.15 ± 0.01^a^	0.12 ± 0.07^a^	Fruity, Apple, Pineapple
1-Butanol, 3-methyl	1.69	0.16 ± 0.01^a^	0.17 ± 0.11^a^	0.15 ± 0.20^a^	0.17 ± 0.05^a^	0.16 ± 0.10^a^	Fusel, Bitter, Balsamic
2-Heptanone	1.53	0.11 ± 0.18^a^	0.12 ± 0.33^a^	0.11 ± 1.01^a^	0.11 ± 1.12^a^	0.00 ± 0.00^b^	Fruity, Cheese, Soapy
2-Pentanone	1.46	0.10 ± 0.17^a^	0.13 ± 0.12^a^	0.12 ± 0.02^a^	0.09 ± 0.05^a^	0.15 ± 0.10^a^	Fruity, Sweet
Camphene	1.45	0.28 ± 0.02^a^	0.23 ± 0.08^b^	0.23 ± 0.11^b^	0.22 ± 0.04^b^	0.21 ± 0.08^b^	Camphor, Pine, Woody
1-phenylethanol	1.37	0.26 ± 0.01^a^	0.01 ± 0.02^d^	0.17 ± 0.01^c^	0.21 ± 0.03^b^	0.01 ± 0.01^d^	Floral, Rose, Honey
1-Propanol, 2-methyl	1.34	0.02 ± 0.08^d^	0.33 ± 0.10^b^	0.02 ± 0.01^d^	0.43 ± 0.10^a^	0.12 ± 0.03^c^	Winey, Fusel, Bitter
Ethanol	1.29	0.10 ± 0.02^c^	0.11 ± 0.02^c^	0.14 ± 0.01^bc^	0.21 ± 0.05^a^	0.18 ± 0.10^ab^	Pungent, Ethereal, Sweet
2-Butanone	1.23	0.16 ± 0.01^c^	0.21 ± 0.01^b^	0.08 ± 0.10^d^	0.15 ± 0.01^c^	0.34 ± 0.04^a^	Sweet, Minty
1- butanol	1.21	0.15 ± 0.04^b^	0.14 ± 0.10^b^	0.12 ± 0.01^b^	0.34 ± 0.05^a^	0.22 ± 0.13^ab^	Medicinal, Fusel, Banana
1-Propanol	1.19	0.15 ± 0.10^a^	0.20 ± 0.20^a^	0.19 ± 0.13^a^	0.18 ± 0.10^a^	0.23 ± 0.03^a^	Pungent, Alcohol, Sweet
3-Methyl-2-butenal	1.04	0.28 ± 0.02^b^	0.09 ± 0.07^d^	0.19 ± 0.14^c^	0.15 ± 0.01^cd^	0.34 ± 0.07^a^	Green, Almond, Malt
ethyl 2-methylbutanoate	0.66	64.66 ± 0.10^b^	61.26 ± 0.02^c^	94.11 ± 0.20^a^	63.81 ± 0.03^b^	40.21 ± 1.01^d^	Fruity, Apple, Strawberry
Butanal	0.68	13.12 ± 0.37^c^	18.57 ± 0.10^a^	12.75 ± 1.00^c^	16.91 ± 0.08^b^	12.04 ± 0.03^c^	Pungent, Chocolate, Green
Ethyl propanoate	0.63	27.86 ± 0.07^c^	33.46 ± 0.11^a^	26.43 ± 0.02^d^	31.05 ± 0.09^b^	25.04 ± 0.20^e^	Fruity, Rum, Pineapple
Thiophene	0.64	32.23 ± 0.71^c^	57.20 ± 0.80^a^	19.48 ± 0.12^e^	42.62 ± 0.08^b^	27.09 ± 0.03^d^	Burnt, Sulfurous, Gasoline
(E, E)-2,4-heptadienal	0.75	7.25 ± 0.31^d^	6.96 ± 0.02^d^	20.26 ± 0.29^a^	8.27 ± 0.10^c^	12.65 ± 0.30^b^	Fatty, Nutty, Deep-fried
2-methylpropyl 2-methylpropanoate	0.84	21.11 ± 0.17^c^	45.84 ± 0.17^a^	21.84 ± 0.20^b^	15.64 ± 0.25^d^	12.71 ± 0.21^e^	Fruity, Apple, Pineapple
2-Methylisoborneol	0.81	16.13 ± 0.10^c^	25.14 ± 0.03^a^	11.21 ± 0.20^d^	20.05 ± 0.17^b^	8.01 ± 0.01^e^	Earthy, Musty, Camphor

SCP3 was characterized as the most intensely fruity sample, evidenced by its highest levels of the key ester OACs (Ethyl 2-methylpropionate and ethyl 2-methylbutanoate), while simultaneously having the lowest levels of less desirable green/pungent notes (e.g., 1-Penten-3-one) (Sanz et al., 2018). In contrast, SCP2 exhibited a greener, more pungent profile with prominent 1-Penten-3-one and subdued fruity esters. SCP5 presented a unique case with the lowest overall fruity ester intensity but distinct elevated levels of 2-Butanone and 3-Methyl-2-butenal. SCP1 was distinguished by its notable floral note from 1-phenylethanol and a unique camphorous note from Camphene, while SCP4 showed pronounced winey and fusel alcohol characteristics from 1-Propanol, 2-methyl and 1-butanol.

The table lists the variable importance in projection (VIP) scores from PLS-DA and the ROAV values (mean ± standard deviation, *n* = 3) across five SCP samples (SCP1-SCP5).

#### 3.2.3 Comprehensive analysis and function-oriented screening of OACs using GC × GC-TOF-MS

A targeted screening strategy was used to functionally classify key flavor compounds into two groups: 1) stable base compounds, and 2) potential differential markers. First, GC × GC-TOF-MS analysis detected 213 VOCs across the five samples, providing precise identification and quantification beyond the range of GC-IMS. Screening was then conducted with the following criteria. Core flavor compounds were defined as those that were ubiquitous (detected in ≥80% of groups), stable in content (CV ≤ 40%), and with a confirmed sensory contribution (OAV ≥ 1). Characteristic differential compounds showed significant variation between samples (p < 0.05) and had actual aroma activity (OAV ≥ 1 in at least one sample).

A set of 27 core OACs was identified as consistently present across all samples. (Table S2), establishing a fundamental chemical basis for their characteristic flavor profile. The profile was dominated by esters, particularly long-chain fatty acid ethyl esters such as ethyl tetradecanoate, ethyl hexadecanoate, and ethyl dodecanoate, which provide a stable aromatic foundation with waxy, creamy, and mild fruity notes (Stanzer et al., 2023). These were complemented by characteristic aroma-active terpenes including linalool (floral, citrus) and α-terpineol (lilac, pine), along with phenylethyl alcohol contributing distinct rose-like floral aromas (Dippong et al., 2023). Notably, several compounds derived from diverse biochemical pathways were universally detected. These included ethyl salicylate (wintergreen), phenol, 4-ethyl-2-methoxy- (smoky, spicy), and 3-Buten-2-one, 4-(2,6,6-trimethyl-1-cyclohexen-1-yl)- (violet, woody) (Füllemann & Steinhaus, 2020). The consistent presence of these compounds, accompanied by low coefficients of variation (CV ≤ 40%), confirmed their role as the essential “flavor backbone” in traditional SCP.

Additionally, 28 differential OACs were identified through the aforementioned screening method (Table S2). These differential compounds exhibited marked distribution variations across regional samples, collectively shaping the distinct sensory identities of products from different origins. Certain compounds showed exclusive presence in specific samples, such as Fenchol and ethyl decanoate detected only in SCP3 (Guizhou), and Caryophyllene and 2-heptanone uniquely found in SCP2 (Hebei), serving as characteristic regional markers. Other compounds, while present in multiple samples, displayed significant quantitative differences (CV > 40%), including Phenol, 2-methoxy-(highest in SCP1), 1-pentanol (most abundant in SCP3 and SCP5), and 2-butanone, 4-(2,6,6-trimethyl-2-cyclohexen-1-yl)-(predominant in SCP4). These differential compounds, spanning esters, terpenes, and phenols, contribute diverse aroma nuances including fruity, floral, woody, and smoky notes. The combination of the stable core compounds and these variable differential compounds created both the consistent fundamental flavor profile and the unique regional characteristics of traditional SCP. In total, 53 significant OACs were identified, comprising 24 esters, 19 terpenes/terpenoids, 10 aromatic compounds, 9 alcohols, 7 ketones, 3 heterocyclic compounds, 2 aldehydes, and 1 acid. Partial list of compounds was shown in Table 2.

**Table 2:** Qualitative and Quantitative Analysis of SCP Using GC × GC-TOF-MS.

Compound Name	CAS	1t_R_(min)	^2^*t*_R_(s)	RI	Concentration[μg/g]				
					SCP1	SCP2	SCP3	SCP4	SCP5
Phenylethyl Alcohol	60–12-8	19.3827	3.444	1075.00	3.60 ± 0.40^b^	1.16 ± 0.12^b^	13.21 ± 5.08^a^	3.90 ± 1.64^b^	4.42 ± 2.31^ab^
Phenol, 4-ethyl-2-methoxy-	2785-89-9	25.3827	3.041	1215.15	30.69 ± 2.92^ab^	7.73 ± 0.77^b^	48.76 ± 2.71^ab^	59.11 ± 2.26^a^	13.74 ± 3.09^b^
Methyl salicylate	119–36-8	22.4494	3.087	1151.43	2.81 ± 0.60^b^	3.89 ± 0.86^ab^	3.42 ± 1.78^ab^	6.80 ± 2.13^a^	2.28 ± 0.98^b^
Linalool	78–70-6	18.8491	2.125	1054.99	4.62 ± 0.34^a^	2.68 ± 0.20^a^	10.13 ± 4.61^a^	9.60 ± 2.68^a^	6.34 ± 3.61^a^
Hexadecanoic acid, ethyl ester	628–97-7	45.3823	2.054	1965.37	63.77 ± 4.27^b^	13.76 ± 1.37^c^	146.28 ± 2.74^a^	64.04 ± 2.95^b^	38.82 ± 1.60^bc^
3-Buten-2-one, 4-(2,6,6-trimethyl-1-cyclohexen-1-yl)-	14,901–07-6	32.0492	2.732	1361.46	1.75 ± 0.71^ab^	0.55 ± 0.78^b^	–	3.33 ± 1.13^a^	0.54 ± 0.23^b^
Pyrazine, 2-methoxy-3-(2-methylpropyl)-	24,683–00-9	21.9165	2.475	1140.01	0.24 ± 0.05^a^	0.13 ± 0.03^ab^	0.29 ± 0.10^a^	–	0.28 ± 0.15^a^
Phenol, 2-methoxy-	90–05-1	18.45	3.268	1040.02	1.07 ± 0.06^a^	0.38 ± 0.10^b^	0.26 ± 0.09^ab^	0.44 ± 0.17^b^	0.07 ± 0.05^c^
Fenchol	1632-73-1	24.0498	2.553	1185.73	–	–	0.21 ± 0.08^a^	–	–
Caryophyllene	87–44-5	30.0499	2.158	1319.57	–	0.13 ± 0.02^a^	–	–	–
2H-Pyran, 3,6-dihydro-4-methyl-2-(2-methyl-1-propenyl)-	1786-08-9	21.5163	2.404	1131.44	–	0.06 ± 0.00^a^	–	–	–
2-Butanone, 4-(2,6,6-trimethyl-2-cyclohexen-1-yl)-	31,499–72-6	29.9165	2.551	1316.77	–	0.04 ± 0.00^b^	–	0.15 ± 0.08^a^	–

1 t_R_(min): first-dimension retention time, ^2^t_R_(s): second-dimension retention time, RI: retention indices for various compounds. SCP1 (Jiangsu), SCP2 (Hebei), SCP3 (Guizhou), SCP4 (Shandong), and SCP5 (Henan).

**Fig. 3:**
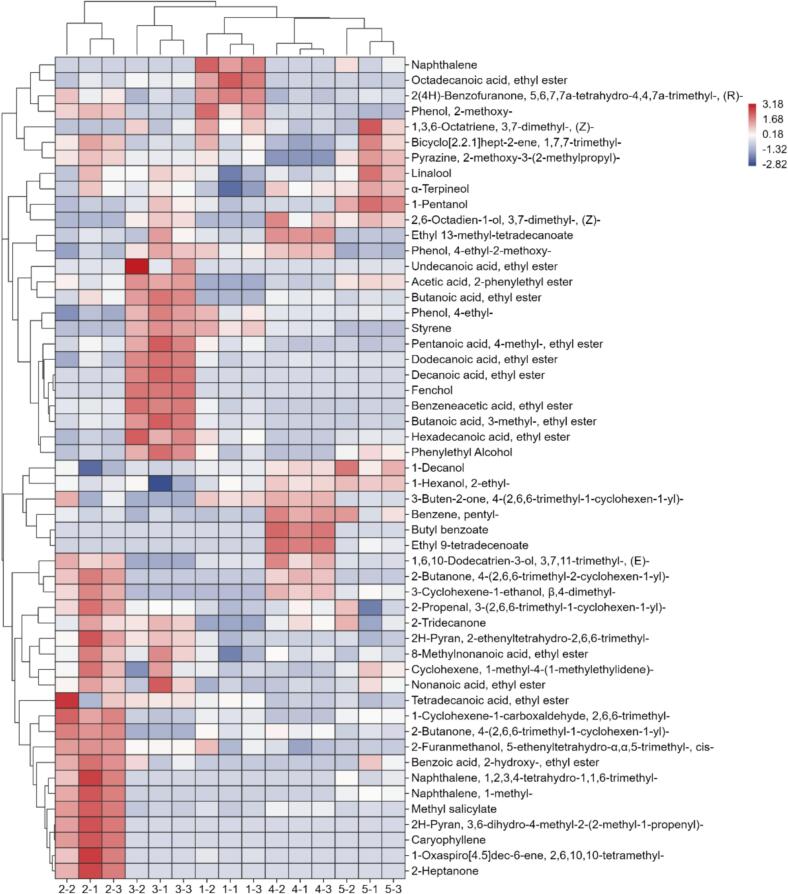
GC × GC-TOF-MS Analysis Heat Map of Five SCPs. Sample codes in the fig. (1–5) represent SCP1 to SCP5, respectively. Each sample has 3 parallel data points.

As shown in Fig. 3, a hierarchical clustering heatmap was constructed to visualize the relative abundance profiles of the significant OACs identified across the five samples. Hierarchical clustering of the samples, as shown in the column dendrogram, reveals a clear and significant grouping pattern. SCP4 and SCP5 form a tight, distinct cluster, indicating a highly similar volatile profile. This clustering aligned with the conclusions drawn from the *E*-nose radar map, where the enclosed areas of SCP4 and SCP5 exhibited a high degree of overlap. However, SCP1, SCP2, and SCP3 do not cluster with each other; each was positioned on a separate branch of the dendrogram, signifying that their overall volatile compositions are unique and distinct.

The hierarchical clustering analysis revealed that SCP4 and SCP5 share a strong similarity in their volatile profiles, as indicated by their tight grouping. This shared chemical signature is defined by the co-enrichment of a specific set of alcohols, which appear as regions of consistently high intensity in the corresponding heatmap. The key compounds contributing to this common foundation include α-Terpineol, which is known for its characteristic floral and lilac-like aroma; (Z)-3,7-dimethyl-2,6-octadien-1-ol (often associated with citrus and rose notes); 1-Decanol, imparting waxy and citrus rind nuances; and 2-ethyl-1-hexanol, which contributes a sweet, rose-like scent. Together, this combination of monoterpenoid and higher alcohols established a foundational aroma profile for the SCP4/SCP5 cluster, characterized by pronounced floral, citrusy, and sweet-waxy notes.

The volatile profiles of SCP1, SCP2, and SCP3 were distinctly differentiated by their dominant compound classes. Specifically, SCP1 was characterized by high levels of naphthalene and 2-methoxyphenol, imparting pungent, smoky, and spicy aromas. Elevated amounts of octadecanoic acid ethyl ester and (Z)-1,3,6-octatriene added waxy-fatty and fresh citrus nuances, thereby creating a profile strongly defined by phenolic and spicy notes. In contrast, SCP2 exhibited a more complex signature dominated by terpenoids such as caryophyllene and various ketones including 2-heptanone, which collectively contribute woody, spicy, and creamy-fruity characteristics (Xi et al., 2023). This complexity was further enhanced by esters like benzoic acid, 2-hydroxy-, ethyl ester, adding sweet and balsamic undertones.

Similarly unique, SCP3 presented a dual-character profile, prominently featuring a suite of fatty acid ethyl esters (e.g., decanoic acid, dodecanoic acid, and tetradecanoic acid ethyl esters) that deliver sweet, waxy, and fruity note. Concurrently, this sample showed a significant presence of phenolic compounds, especially phenol, 4-ethyl-2-methoxy-(4-ethylguaiacol), which provides distinct smoky and clove-like aromas associated with fermentation.

#### 3.2.4 Evaluation of multi-platform concurrent strategies: Complementarity analysis of GC-IMS and GC × GC-TOF-MS in flavor profiling

To evaluate the applicability and complementarity of different analytical techniques for resolving complex flavor systems, this study integrated and compared the analytical results obtained from GC-IMS and GC × GC-TOF-MS. Comparing the OACs identification results from the two technical platforms revealed that their complementarity far outweighs their overlap. While GC × GC-TOF-MS identified 53 characteristic OACs dominated by esters and terpenes, GC-IMS detected 23 OACs primarily consisting of alcohols and aldehydes. Critically, only a single compound, 2-heptanone, was consistently identified by both platforms.

This remarkably low overlap is not indicative of analytical inconsistency but rather reflects the fundamental differences in the operational principles and detection capabilities of the two techniques. GC × GC-TOF-MS, with its superior separation power and high mass accuracy (Jiang et al., 2015), excels at resolving complex mixtures of mid-to-high VOCs (Nolvachai et al., 2017), particularly esters (e.g., ethyl tetradecanoate, ethyl hexadecanoate) and terpenes (e.g., linalool, α-terpineol) that characterize the fermented, fruity-floral aroma profile. These compounds often have higher molecular weights and longer retention times.

In contrast, GC-IMS demonstrates exceptional sensitivity toward highly volatile, low molecular weight compounds and isomeric molecules that are challenging to capture by conventional GC–MS (Gu et al., 2021). The GC-IMS profile was characterized by short-chain alcohols (e.g., 1-butanol, 2-methyl-1-propanol), aldehydes (e.g., hexanal, butanal), and ketones (e.g., 2-butanone, 2-pentanone), which contribute fresh, green, and pungent notes. Many of these highly VOCs are either below the detection limit of GC × GC-TOF-MS or elute too quickly to be effectively trapped and focused by the SPME fiber used in the GC × GC-TOF-MS sample preparation (Nolvachai et al., 2023).

The complementary nature of these techniques is further evidenced by their coverage of different chemical spaces. GC-IMS reveals the fundamental backbone of esters and alcohols, while GC × GC-TOF-MS further delineates a more complete and refined flavor profile-an ester-based fruity foundation blended with terpenoid floral and woody notes. Both findings collectively demonstrate that the flavor profile of SCP is a complex interplay between the aromas produced during fermentation and the inherent characteristics of the raw ingredients. The sole overlapping compound, 2-heptanone (blue-cheese, fruity), possesses intermediate volatility and a molecular structure amenable to detection by both systems, serving as a validation point for the reliability of both analytical approaches.

In conclusion, the minimal overlap between the two techniques underscores their orthogonal nature. GC-IMS provides a rapid snapshot of the highly volatile aroma compounds, while GC × GC-TOF-MS delivers a deep dive into the less volatile but often more aroma-impactful compounds. This multi-platform approach is therefore essential for constructing a comprehensive flavor blueprint of complex fermented foods like SCP.

#### 3.2.5 Biochemical origins and formation mechanisms of key flavor compounds

The aroma differences among SCP samples from different geographical origins are attributed to variations in biochemical pathways during fermentation, influenced by raw materials and microbial communities. SCP3 showed the highest levels of fruity esters (e.g., ethyl tetradecanoate). These esters are synthesized through esterification reactions catalyzed by microbial esterases. The abundance suggests active ester-producing microorganisms or favorable fermentation conditions. SCP3 also had elevated phenolic compounds (e.g., 4-ethylguaiacol), which are formed via microbial decarboxylation of phenolic acids. The co-occurrence indicates complex microbial metabolism. SCP4 and SCP5 clustered together with similar volatile profiles characterized by terpenoids (e.g., α-terpineol) and higher alcohols. Terpenoids can originate from both raw materials and microbial transformation during fermentation. Higher alcohols are often byproducts of yeast metabolism. SCP1 was distinguished by phenolic compounds (e.g., 2-methoxyphenol), which may derive from specific microbial activities or raw material characteristics. SCP2 (Hebei) presented a complex signature with terpenoids and ketones, suggesting diverse microbial metabolism or unique fermentation conditions.

The geographical variation in aroma profiles reflects differences in biochemical pathways and microbial activities. Raw material composition, fermentation conditions, and indigenous microbial communities collectively shape the volatile flavor characteristics of each sample.

### 3.3 Analysis of non-volatile metabolites in SCP using UHPLC-MS/MS

#### 3.3.1 Comprehensive analysis

For mass spectrometry-based metabolomics research, QC samples are necessary to ensure reliable and high-quality metabolomics data. The relative standard deviation (RSD) distribution of features in QC samples is applied to assess the repeatability of QC samples. A lower RSD means better instrument stability. As shown in Fig. S2, over 80% of the total peaks in the experimental QC samples had an RSD ≤ 30%. This result proved good system stability, so the data was suitable for subsequent analysis.

**Fig. 4:**
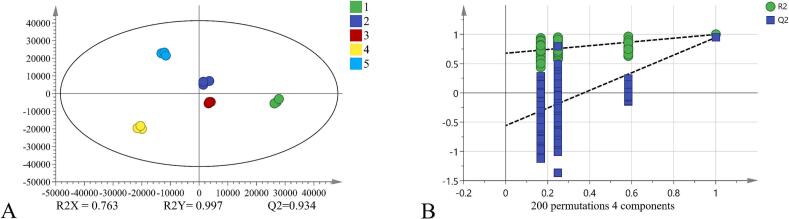
OPLS-DA score plot (A) and permutation test results (B) for five SCPs. Sample codes in the fig. (1–5) represent SCP1 to SCP5, respectively.

OPLS-DA was used for differential screening of non-volatile metabolites in five SCPs. As displayed in Fig. 4A, the three parallel replicates of each sample clustered closely, which showed good data reproducibility. The model evaluation parameters R^2^X, R^2^Y, and Q^2^ were 0.763, 0.997, and 0.934, respectively. A Q^2^ value greater than 0.5 indicates good model interpretability and predictive ability. To prevent overfitting, a univariate permutation test (Fig. 4B) was used to evaluate the modeling performance of OPLS-DA, and the results were further verified with 200 random permutations. The intersection of the Q^2^ regression line and the vertical axis was less than 0, which meant the model had no overfitting and was valid. Thus, this result was appropriate for the identification and analysis of non-volatile metabolites in different SCPs. Based on preset *P*-values (*P* < 0.05) and VIP scores (VIP > 1.0), 49 umami-related differential metabolites (Table S3) were identified. The screening and validation process used metabolic databases, mass spectrometry fragment information, and literature data.

#### 3.3.2 Differential analysis of non-volatile metabolite composition in SCP

**Fig. 5:**
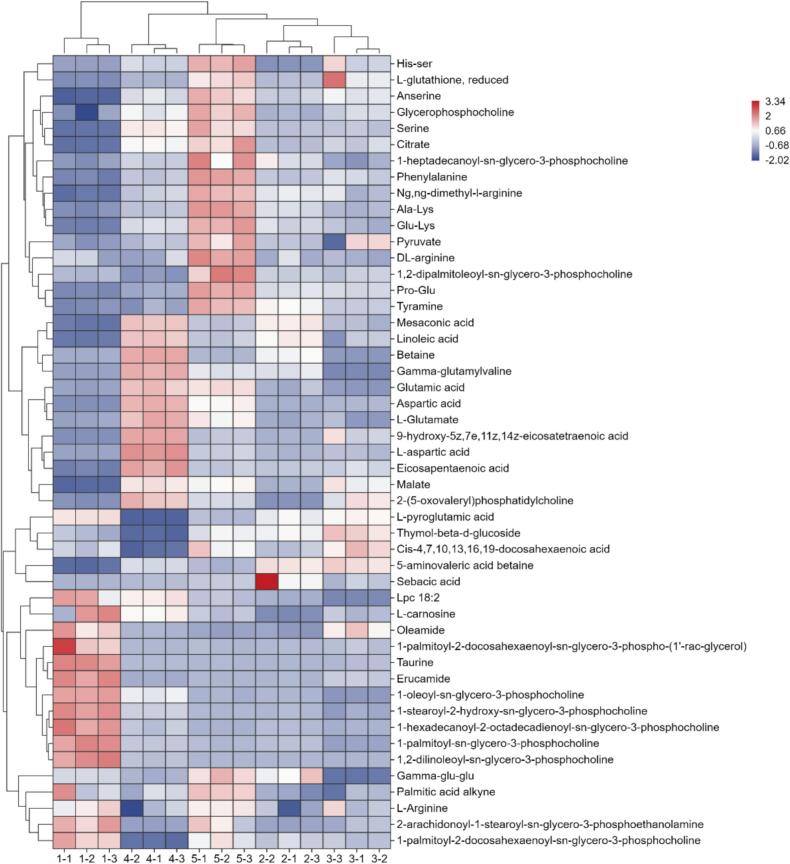
Clustering Heatmap of Distinct Flavor Compounds in Five SCPs. Sample codes in the fig. (1–5) represent SCP1 to SCP5, respectively. Each sample had 3 parallel data points.

Further analysis was performed on the 49 identified umami-related differential metabolites. These metabolites belonged to multiple biochemical categories. Specifically, they included 11 amino acids, 20 lipids, 8 organic acids, 7 peptides, 1 aromatic monoamine metabolite, 1 phenolic glycoside compound, and 1 quaternary ammonium salt derivative (Table S3). The relative abundance of these metabolites was shown in a heatmap (Fig. 5). This abundance distribution might have reflected the metabolic differences between different SCPs. Based on the 49 metabolites, the SCPs were clustered into five groups. This grouping result corresponded precisely with the geographical origins of the raw materials: each cluster represented SCP sourced from a distinct region. As shown in Fig. 5, different SCPs share some similar non-volatile umami metabolites. However, notable differences still existed between them. These differential metabolites were mainly umami peptides and umami amino acids. The umami peptides included Pro-Glu, Glu-Lys, Lys-Gly, and γ-glutamyl peptides. The umami amino acids included glutamic acid and aspartic acid.

#### 3.3.3 Identification of umami components in SCP

Umami is one of the characteristic factors influencing the development of food flavor. The taste mechanism of umami is complex, as it is associated with both the basic umami substances in food and the interactions among internal VOCs (Kurihara, 2015). In recent years, glutamic acid, aspartic acid, succinic acid, and oligopeptides are recognized as the primary umami compounds in foods (Istiqamah et al., 2019). In this study, *E*-tongue results (Fig. 1) showed that SCP exhibited a pronounced umami profile. Non-targeted metabolomics identification of flavor metabolites (Table S3) corroborated the E-tongue findings, and the results indicated that the umami components of SCP primarily include amino acids and their derivatives, peptides, and lipids. Heatmap analysis of the key umami-related metabolites revealed distinct compositional profiles among the five SCPs (Fig. 5).

Glutamic acid and aspartic acid are the primary contributors to umami in most natural sources (Chen et al., 2021). The substantially higher levels of these two amino acids in SCP4 likely contribute to its stronger umami perception. This distinct profile strongly supports the superior umami-enhancing potential of SCP4 in the five samples. Additionally, the levels of bitter amino acids (e.g., phenylalanine) in SCP5 were significantly higher than in the other SCPs. In contrast, the lower concentrations observed in the other four SCPs, according to literature, fall within a range that could enhance umami perception rather than contribute to bitterness (Lioe et al., 2005). Moreover, betaine was found at elevated levels specifically in SCP4. Given its established use in Japan to mimic seafood flavor and its known umami-enhancing property (Chen et al., 2021), this compound is therefore inferred to be a key contributor to the distinct umami characteristics of SCP4 in the present study.

Pro-Glu was previously isolated as an umami peptide from hydrolyzed chicken protein (Maehashi et al., 1999); and glutamate-rich peptides such as Glu-Lys were known umami contributors in fish protein hydrolysates (Noguchi et al., 1975). In the present study, both Pro-Glu and Glu-Lys were found at markedly higher levels in SCP5 compared to the other SCPs, which could contribute to a stronger umami perception. Moreover, SCP5 was distinguished by its uniquely high reduced L-glutathione content. Given its reported role in enhancing umami perception (Goto et al., 2016), this likely contributed a unique modulatory influence to SCP5's taste profile. Additionally, the notably high level of γ-glutamylvaline in SCP4 was of particular interest, as this peptide class was known to activate the oral CaSR, imparting kokumi attributes and enhancing umami (Brennan et al., 2014). This implied a functional role for γ-glutamylvaline in shaping the complex umami profile of SCP4.

Aldehydes like (E,E)-2,4-decadienal, potential products of lipid oxidation in salted chilis, can synergistically enhance umami (Mouritsen et al., 2019), as evidenced in systems like fermented bean curd (He et al., 2020). Given the concomitantly higher levels of aldehyde-precursor lipids (e.g., linoleic acid derivatives) in SCP1 and SCP4, we inferred a greater generation of lipid-derived aldehydes in these samples, which would thereby enhance their umami intensity. The detection of glycerophospholipids in SCP is functionally noteworthy. Similar lipids were known to regulate the release of key volatile flavor compounds (Ahmmed et al., 2023), and lipids could also suppress the oral release of bitter substances (Banerjee et al., 2021), thereby preventing bitterness from masking umami. Given this functional context, the significantly higher abundance of these glycerophospholipids in SCP5 is particularly suggestive. It leads to the hypothesis that in SCP5, these enriched lipid metabolites (including glycerophospholipids, fatty acids, and their oxidation products) synergize with lipid-derived volatiles to significantly enhance umami perception.

The five SCPs exhibited distinct profiles of umami-related compounds. Notably, despite SCP5's complex and abundant umami metabolites, it showed a lower umami intensity by electronic tongue, likely due to suppression by its higher content of bitter and astringent substances.

#### 3.3.4 Correlation analysis between key non-volatile metabolites and sensory profiles

**Fig. 6:**
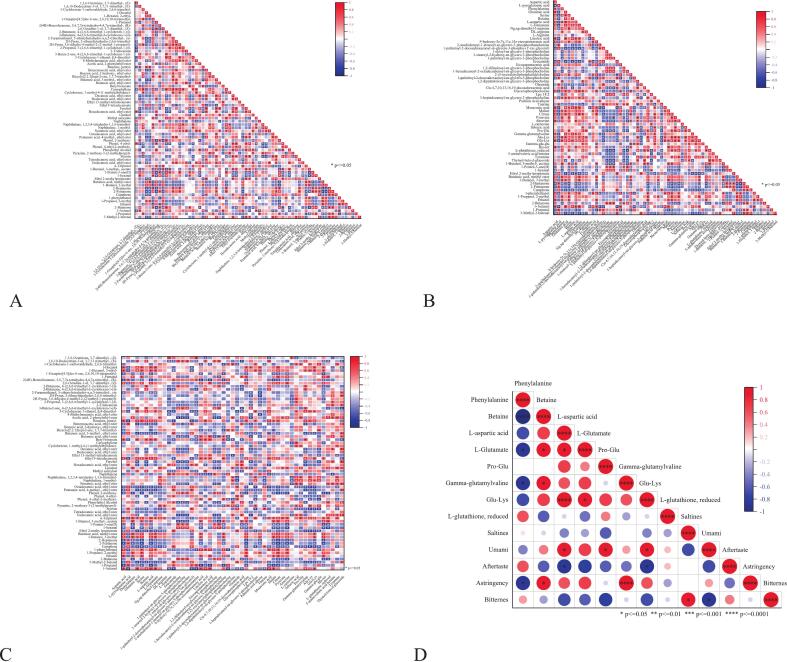
Correlation Analysis Heatmap. (A) Correlation analysis between OACs and OACs;(B) Correlation analysis between OACs and non-volatile metabolites; (C) Correlation analysis between non-volatile metabolites and non-volatile metabolites; (D) Correlation analysis between key non-volatile metabolites and sensory attributes.

Correlation analysis identified the key quantitative drivers of SCP taste (Fig. 6D). Notably, umami intensity, as quantified by our sensory panel (see the sensory profile radar chart in supplementary Fig. S3), showed significant positive correlations with L-aspartatic acid and the umami peptides Pro-Glu and Glu-Lys, thereby quantitatively confirming their role as key taste-active compounds, as established in earlier sections.

More importantly, the analysis revealed a fundamental metabolic antagonism central to taste balance. A strong negative correlation was observed between key umami-enhancing compounds (L-glutamate and betaine) and the bitter amino acid phenylalanine (betaine-phenylalanine: *p* < 0.0001). This antagonism provides a direct biochemical rationale for the taste profile of SCP5 described previously: its high abundance of umami peptides was effectively counterbalanced by a concurrently high concentration of bitter compounds. Furthermore, a significant negative correlation between the sensory attributes of bitterness and umami (*p* < 0.05) provided additional support for this interpretation.

Taken together, these quantitative relationships demonstrate that the final taste perception is governed not by individual metabolites in isolation, but by the net balance of competing taste pathways. This insight shifts the focus from merely identifying taste compounds to understanding their functional balance, thereby providing clear targets for the targeted optimization of SCP taste.

### 3.4 Comprehensive correlation analysis of flavor compounds in SCP

The Spearman correlation analysis elucidated the complex interaction network among 75 OACs (23 from GC-IMS and 53 from GC × GC-TOFMS) and 49 non-volatile metabolites.

The aroma structure was detailed in the volatile-volatile map (Fig. 6A). The heatmap revealed strong positive correlations (highly red squares) among certain OACs, suggesting they may synergistically enhance flavor perception. Esters and terpenes exhibited such synergy: for example, esters like ethyl hexanoate (fruit-like) positively correlated with terpenes like linalool (floral). This “fruity-floral” combination created a more intense and harmonious aroma profile than individual compounds, enhancing flavor richness at the olfactory level and indirectly contributing to a “freshness-enhancing” effect (Holt et al., 2019).

Simultaneously, OACs and non-volatile metabolites exhibited cross-modal synergistic umami enhancement (Fig. 6B). Glutamic acid showed strong positive correlations with ethyl 2-methylpropanoate and 1-butanol, consistent with the umami-enhancing effects of volatiles like 2,6-dimethylpyrazine in soy sauce systems. These esters and alcohols bind to the volatile-binding domain of the T1R1/T1R3 umami receptors, inducing conformational changes that amplify glutamate perception, thereby achieving cross-modal “umami-alcohol” enhancement (Hao et al., 2024). Heat maps also revealed a strong positive correlation between aspartic acid and β-phenylethanol. As a typical umami amino acid, aspartic acid enhances the fundamental savory perception of foods; β-phenylethanol contributes a rose-like floral note and can intensify overall aroma strength through cross-modal interactions (Zhang et al., 2019). Thus, in SCP, the coexistence of these compounds achieved synergistic umami enhancement-providing a savory foundation while elevating the flavor profile's complexity and perceived intensity through floral notes. Glutamic acid and other compounds were positively correlated with certain furan derivatives (such as furfural, which imparts a toasty sweet aroma) or phenyl derivatives (such as phenethyl alcohol, which imparts a rose-like fragrance). This indicated that while tasting umami, the nasal cavity simultaneously perceives these pleasant aromas. The brain integrates this composite signal of “umami + caramel/floral notes” into a more intense, fuller perception of “rich savory flavor” (Hou et al., 2022; Lee et al., 2006; Rolls & Baylis, 1994).

In Fig. 6C, γ-glutamylpeptide showed a strong positive correlation with L-glutamic acid. Studies have confirmed that γ-glutamylpeptide exhibits significant synergistic umami-enhancing effects with L-glutamic acid. Both substances enhance intracellular calcium ion release by jointly activating the T1R1/T1R3 umami receptors (Yang et al., 2021). In summary, the strong correlation between non-volatile metabolites and OACs in SCP revealed a multi-level, cross-modal synergistic flavor-enhancing network. This provided clear molecular targets for further optimizing fermentation processes and enhancing product nose quality.

## 4 Conclusion

This study systematically characterized the flavor profiles of five regionally distinct SCPs using multiple analytical techniques, including *E*-nose, E-tongue, GC-IMS, GC × GC-TOF-MS, and UHPLC-MS/MS. The results confirmed significant differences in the overall flavor profiles among the five SCPs. GC-IMS and GC × GC-TOF-MS demonstrated high complementarity in flavor analysis: GC-IMS enabled rapid trace detection, while GC × GC-TOF-MS provided in-depth precise identification. A total of 75 characteristic OACs were screened. The volatile profiles of the five SCPs were dominated by rich combinations of fatty, fruity, and phenolic-spicy notes. Notably, SCP3 from Guizhou exhibited the strongest overall aroma intensity, characterized by a potent ester-phenol profile that delivered a complex combination of sweet-fatty richness and fermented, smoky-phenolic character. This unique profile stems from the active microbial pathways for both esterification and phenolic acid decarboxylation identified in this sample. Non-targeted metabolomics analysis elucidated that the pronounced umami originated from abundant non-volatile metabolites, including amino acids, peptides, and lipids. The heatmap revealed significant differences in umami-related compounds among the five SCPs: SCP1 was enriched in lipids, SCP4 in umami amino acids and their derivatives, and SCP5 in peptides. Building on this, correlation analysis between metabolites and sensory profiles quantitatively revealed that the final taste perception of SCP is influenced not by individual compounds, but by synergistic (e.g., aspartate and umami peptides) and antagonistic (e.g., glutamate/betaine and phenylalanine) interactions among them. Furthermore, correlation analysis demonstrated significant synergistic effects between key aroma compounds and umami-related substances, explaining their “savory-aromatic” fusion at the molecular level. These findings demonstrate that geographical origin significantly influenced the flavor profiles of SCP. Since all samples were derived from the same chili variety and processed under identical conditions, the distinct flavor compounds identified across the five regions can be attributed primarily to differences in their geographical origins. This highlights how factors such as local climate, soil, and cultivation practices shape the unique chemical composition, thereby directly influencing the final flavor of the product. Furthermore, synergistic effects between key flavor compounds were observed, contributing to the complexity and enhancement of both aroma and umami perception. This study provides a molecular basis for flavor quality control and targeted flavor enhancement in the industrial production of SCP.

Ethical statement.

The SCP samples were directly evaluated by the trained sensory panelists. Panelists were instructed to assess the taste characteristics and rate the intensity of each attribute using a scale from 0 (not perceived) to 9 (extremely strong). The detailed sensory evaluation procedure is described in Section 2.6.3. All participants were informed of their right to decline participation or to withdraw their consent at any stage without facing any negative consequences. While there was no formal ethics committee available, the research followed the principles and guidelines set forth in the Helsinki Declaration to ensure the ethical treatment of participants.

## CRediT authorship contribution statement

**Wen-ke Yang:** Writing – review & editing, Writing – original draft, Visualization, Formal analysis, Conceptualization. **Rui Deng:** Writing – review & editing, Writing – original draft, Visualization, Formal analysis, Conceptualization. **Ya-qin Huang:** Writing – review & editing. **Hui-ping Xia:** Supervision. **Ke-yao Wang:** Resources. **Yi Zhou:** Funding acquisition. **Ming-xi Liao:** Funding acquisition. **Qing-ming Li:** Writing – review & editing.

## Funding

This work was supported by the Engineering Research Center for Pepper Deep Processing of Hunan Province and Hunan Agricultural University Innovation Foundation For Postgraduate (2024XKCB025).

## Declaration of competing interest

The authors declare that they have no known competing financial interests or personal relationships that could have appeared to influence the work reported in this paper.

## Data Availability

Data will be made available on request.

## Appendix A Supplementary data

Supplementary material Image 1
