# Supplementary material for: Comprehensive profiling of volatile aroma compounds and non-volatile metabolites in five salted chili peppers using multi-omics technologies: E-nose, E-tongue, GC-IMS, GC × GC-TOF-MS, and UHPLC-MS/MS
Source: Food Chem X. 2026 Mar 4;35:103714. doi: 10.1016/j.fochx.2026.103714 (PMC12992073; doi:10.1016/j.fochx.2026.103714)
Supplement: Supplementary file 1 — Supplementary material [file mmc1.docx]

| **A**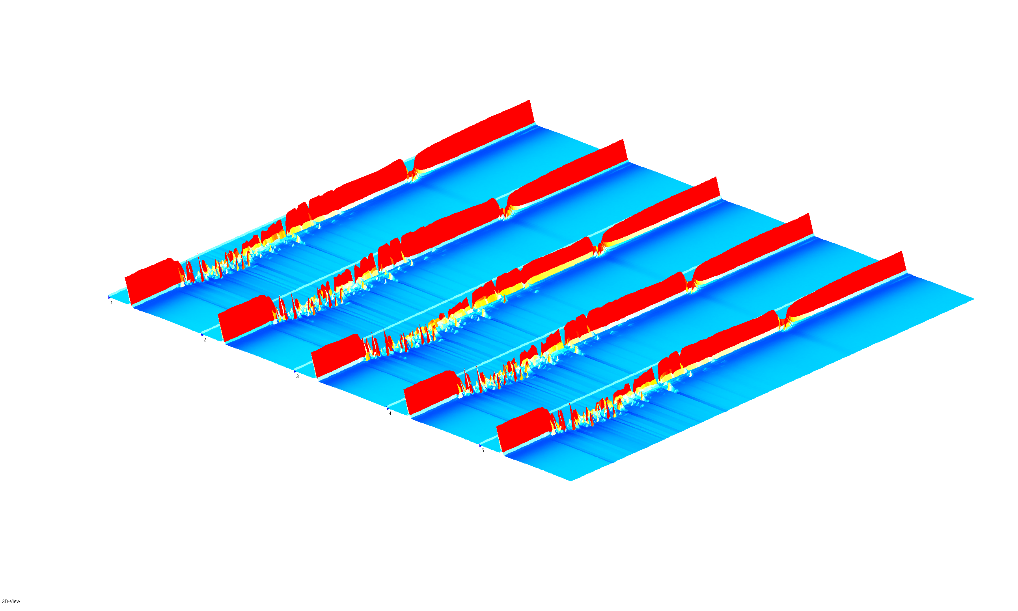 |
| --- |
| **B**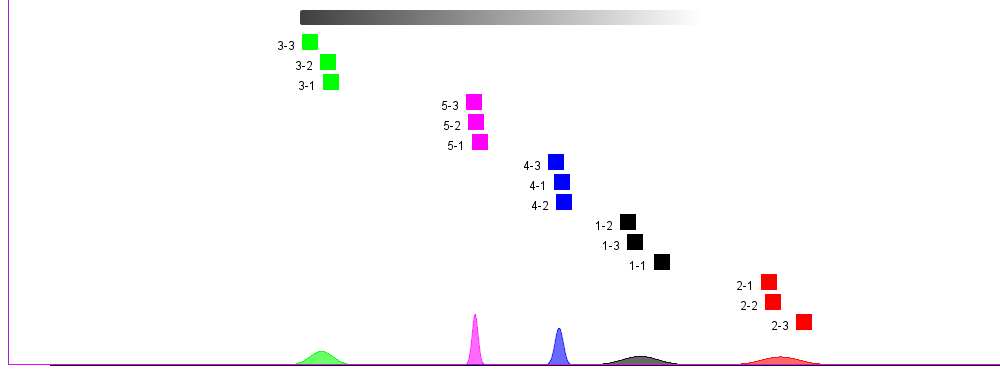 |

**Fig. S1.** (A) GC-IMS three-dimensional spectra of volatile organic compounds from different samples (retention time, migration time, and peak intensity); (B) “nearest neighbor” fingerprint analysis diagrams.

| A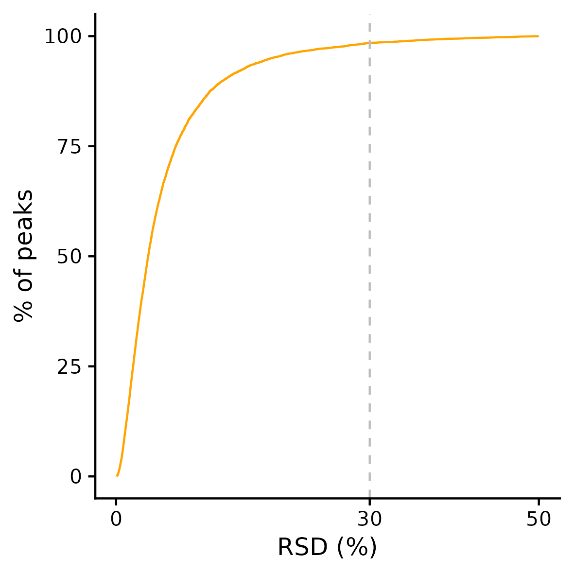 | B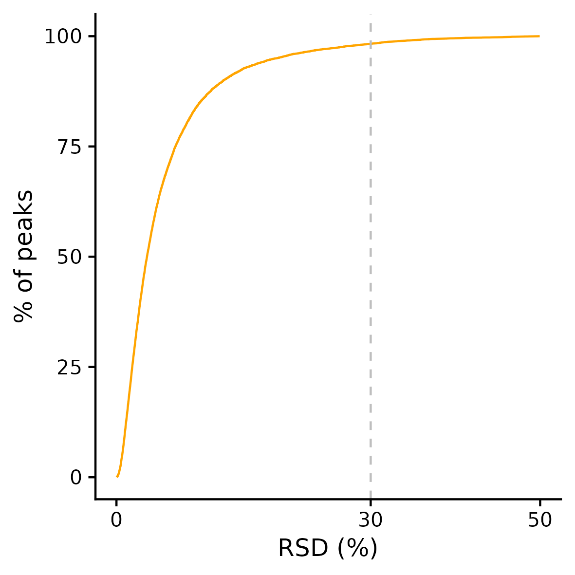 |
| --- | --- |

**Fig. S2.** Relative Deviation of QC Samples in Positive Ion Mode (A); Relative Deviation of QC Samples in Negative Ion Mode (B)

**Table S1** GC-IMS global area set integration parameters obtained from five samples.

| Count | Compound | CAS | Formula | MW^a^ | RI^b^ | Rt^c^[s] | Dt ^d^[ms] | Comment |
| --- | --- | --- | --- | --- | --- | --- | --- | --- |
| 1 | Acetic acid | C64197 | C2H4O2 | 60.1 | 1486.6 | 1252.59 | 1.05031 | D |
| 2 | Acetic acid | C64197 | C2H4O2 | 60.1 | 1484.1 | 1245.623 | 1.16177 | M |
| 3 | Ethyl 2-hydroxypropanoate | C97643 | C5H10O3 | 118.1 | 1352.7 | 923.565 | 1.14355 | D |
| 4 | Ethyl 2-hydroxypropanoate | C97643 | C5H10O3 | 118.1 | 1351.1 | 920.125 | 1.53614 | M |
| 5 | 2-Butanone, 3-hydroxy | C513860 | C4H8O2 | 88.1 | 1292 | 804.542 | 1.06744 | D |
| 6 | 2-Butanone, 3-hydroxy | C513860 | C4H8O2 | 88.1 | 1292.4 | 805.23 | 1.32783 | M |
| 7 | ethyl 2-methylbutanoate | C7452791 | C7H14O2 | 130.2 | 1065.5 | 432.41 | 1.65075 | null |
| 8 | 1-hexanal | C66251 | C6H12O | 100.2 | 1083.1 | 454.639 | 1.26457 | null |
| 9 | 2-Pentanol | C6032297 | C5H12O | 88.1 | 1135.8 | 539.553 | 1.21589 | D |
| 10 | 2-Pentanol | C6032297 | C5H12O | 88.1 | 1134.1 | 536.441 | 1.44051 | M |
| 11 | 2-Heptanone | C110430 | C7H14O | 114.2 | 1192.5 | 653.809 | 1.26457 | D |
| 12 | 2-Heptanone | C110430 | C7H14O | 114.2 | 1193.4 | 655.143 | 1.63305 | M |
| 13 | 1- butanol | C71363 | C4H10O | 74.1 | 1159.6 | 584.97 | 1.18786 | D |
| 14 | 1- butanol | C71363 | C4H10O | 74.1 | 1157.7 | 581.175 | 1.37813 | M |
| 15 | 1-Penten-3-ol | C616251 | C5H10O | 86.1 | 1174.3 | 614.855 | 0.93891 | null |
| 16 | 1-Butanol, 3-methyl | C123513 | C5H12O | 88.1 | 1223.4 | 697.395 | 1.25187 | D |
| 17 | 1-Butanol, 3-methyl | C123513 | C5H12O | 88.1 | 1220.1 | 692.652 | 1.49015 | null |
| 18 | Ethanol | C64175 | C2H6O | 46.1 | 936.5 | 311.57 | 1.04134 | null |
| 19 | 2-Butanone | C78933 | C4H8O | 72.1 | 914.5 | 296.816 | 1.24537 | null |
| 20 | Butanal | C123728 | C4H8O | 72.1 | 839.1 | 251.394 | 1.11304 | null |
| 21 | Heptaldehyde | C111717 | C7H14O | 114.2 | 1169.9 | 605.788 | 1.32661 | null |
| 22 | 1-Pentanol | C71410 | C5H12O | 88.1 | 1261.2 | 754.586 | 1.25269 | null |
| 23 | 2-Pentanone | C107879 | C5H10O | 86.1 | 999.4 | 358.254 | 1.3661 | null |
| 24 | 2-butanol | C78922 | C4H10O | 74.1 | 1008.9 | 368.038 | 1.14819 | null |
| 25 | Ethyl 2-methy lpropionate | C97621 | C6H12O2 | 116.2 | 980.9 | 343.601 | 1.5631 | null |
| 26 | 1-Butanol, 3-methyl-, acetate | C123922 | C7H14O2 | 130.2 | 1135.8 | 539.6 | 1.30398 | D |
| 27 | 1-Butanol, 3-methyl-, acetate | C123922 | C7H14O2 | 130.2 | 1138.7 | 544.937 | 1.75074 | M |
| 28 | alpha-Pinene | C80568 | C10H16 | 136.2 | 1017.9 | 377.611 | 1.28778 | null |
| 29 | Acetic acid ethyl ester | C141786 | C4H8O2 | 88.1 | 898.4 | 286.498 | 1.3433 | null |
| 30 | Ethyl propanoate | C105373 | C5H10O2 | 102.1 | 971.1 | 336.238 | 1.45861 | M |
| 31 | 1,4-Dioxan | C123911 | C4H8O2 | 88.1 | 1097.4 | 473.584 | 1.13355 | null |
| 32 | 1-Propanol, 2-methyl | C78831 | C4H10O | 74.1 | 1110.5 | 495.037 | 1.16985 | D |
| 33 | 1-Propanol, 2-methyl | C78831 | C4H10O | 74.1 | 1106.5 | 488.47 | 1.36879 | M |
| 34 | Cyclooctanol | C696719 | C8H16O | 128.2 | 1176.5 | 619.458 | 1.13266 |  |
| 35 | Thiophene | C110021 | C4H4S | 84.1 | 1022.3 | 382.328 | 1.03922 |  |
| 36 | butylcyclohexane | C1678939 | C10H20 | 140.3 | 1078.7 | 449.029 | 1.0489 |  |
| 37 | 1-Propanol | C71238 | C3H8O | 60.1 | 1057.8 | 423.06 | 1.11822 |  |
| 38 | 4-Methyl-2-pentanone | C108101 | C6H12O | 100.2 | 1017.1 | 376.729 | 1.47753 |  |
| 39 | Ethyl propanoate | C105373 | C5H10O2 | 102.1 | 948.1 | 319.617 | 1.14777 | D |
| 40 | 1-phenylethanol | C98851 | C8H10O | 122.2 | 1052.6 | 416.817 | 1.56112 |  |
| 41 | 1-Penten-3-one | C1629589 | C5H8O | 84.1 | 1042.8 | 405.363 | 1.07992 | D |
| 42 | 1-Penten-3-one | C1629589 | C5H8O | 84.1 | 1038.8 | 400.72 | 1.31235 | M |
| 43 | (E)-2-Pentenal | C1576870 | C5H8O | 84.1 | 1147.6 | 561.548 | 1.10603 | D |
| 44 | (E)-2-Pentenal | C1576870 | C5H8O | 84.1 | 1147 | 560.499 | 1.36251 | M |
| 45 | (E, E)-2,4-heptadienal | C4313035 | C7H10O | 110.2 | 1027.7 | 388.304 | 1.61862 |  |
| 46 | 3-Methyl-2-butenal | C107868 | C5H8O | 84.1 | 1194.2 | 656.167 | 1.36217 |  |
| 47 | Camphene | C79925 | C10H16 | 136.2 | 1085.6 | 457.921 | 1.65718 |  |
| 48 | 2-methylpropyl 2-methylpropanoate | C97858 | C8H16O2 | 144.2 | 1092.1 | 466.493 | 1.80891 |  |
| 49 | 2-Methylisoborneol | C2371428 | C11H20O | 168.3 | 1160.6 | 587 | 1.26202 |  |
| 50 | Acetic acid propyl ester | C109604 | C5H10O2 | 102.1 | 956.1 | 325.315 | 1.47631 |  |
| 51 | Butanoic acid, methyl ester | C623427 | C5H10O2 | 102.1 | 990.2 | 350.647 | 1.14867 | D |
| 52 | Butanoic acid, methyl ester | C623427 | C5H10O2 | 102.1 | 999.7 | 358.487 | 1.44064 |  |

^a-MW means the molecule weight of the volatiles. b-RI means the retention index of the volatiles on capillary column. c-RT means the retention time of the volatiles on GC-IMS. d-DT means the drift time of the volatiles on GC-IMS.^

**Table S2** Concentrations of selected volatile compounds in five SCPs as determined by GC×GC-TOF-MS.

| Compound  Name | CAS | ^1^tR(min) | ^2^tR(s) | RI | Concentration[μg/g] | | | | | Group | Odor description |
| --- | --- | --- | --- | --- | --- | --- | --- | --- | --- | --- | --- |
|  |  |  |  |  | S1 | S2 | S3 | S4 | S5 |  |  |
| α-Terpineol | 98-55-5 | 22.32 | 2.52 | 1148.57 | 2.59±0.31^a^ | 3.29±2.42^a^ | 6.06±2.82^a^ | 7.68±2.19^a^ | 3.88±1.70^a^ | Core | Lilac, Citrus |
| Tetradecanoic acid, ethyl ester | 124-06-1 | 40.45 | 2.03 | 1640.03 | 11.69±3.47^ab^ | 1.94±0.33^b^ | 17.13±5.95^a^ | 12.04±5.43^ab^ | 5.17±0.51^ab^ | Core | Waxy, Creamy |
| Phenylethyl Alcohol | 60-12-8 | 19.38 | 3.44 | 1075.00 | 3.60±0.40^b^ | 1.16±0.12^b^ | 13.21±5.08^a^ | 3.90±1.64^b^ | 4.42±2.31^ab^ | Core | Rose, Honey |
| Phenol, 4-ethyl-2-methoxy- | 2785-89-9 | 25.38 | 3.04 | 1215.15 | 30.69±2.92^ab^ | 7.73±0.77^b^ | 48.76±2.71^ab^ | 59.11±2.26^a^ | 13.74±3.09^b^ | Core | Smoky, Spicy |
| Phenol, 4-ethyl- | 123-07-9 | 21.25 | 3.03 | 1125.71 | 6.09±1.29^ab^ | 1.08±0.04^b^ | 11.44±5.46^a^ | 8.17±3.01^ab^ | 3.71±1.40^b^ | Core | Phenolic, Smoky |
| Pentanoic acid, 4-methyl-, ethyl ester | 25415-67-2 | 13.65 | 2.09 | 941.91 | 0.09±0.01^b^ | 0.03±0.01^b^ | 0.41±0.25^a^ | 0.02±0.02^b^ | 0.01±0.01^b^ | Core | Fruity, Apple |
| Nonanoic acid, ethyl ester | 123-29-5 | 24.58 | 2.01 | 1197.14 | 0.52±0.05^a^ | 0.66±0.27^a^ | 1.92±2.34^a^ | 25.14±3.62^a^ | 0.20±0.09^a^ | Core | Waxy, Fruity |
| Methyl salicylate | 119-36-8 | 22.45 | 3.09 | 1151.43 | 2.81±0.60^b^ | 3.89±0.86^ab^ | 3.42±1.78^ab^ | 6.80±2.13^a^ | 2.28±0.98^b^ | Core | Wintergreen |
| Linalool | 78-70-6 | 18.85 | 2.13 | 1054.99 | 4.62±0.34^a^ | 2.68±0.20^a^ | 10.13±4.61^a^ | 9.60±2.68^a^ | 6.34±3.61^a^ | Core | Floral, Lavender |
| Hexadecanoic acid, ethyl ester | 628-97-7 | 45.38 | 2.05 | 1965.37 | 63.77±4.27^b^ | 13.76±1.37^c^ | 146.28±2.74^a^ | 64.04±2.95^b^ | 38.82±1.60^bc^ | Core | Waxy, Creamy |
| Ethyl 13-methyl-tetradecanoate | 35059-50-8 | 42.05 | 1.99 | 1911.27 | 0.91±0.31^a^ | 0.34±0.05^a^ | 2.05±2.00^a^ | 3.85±1.84^a^ | 2.16±2.56^a^ | Core | Waxy, Fruity |
| Dodecanoic acid, ethyl ester | 106-33-2 | 35.12 | 1.98 | 1420.45 | 4.64±1.67^ab^ | 1.52±0.04^b^ | 10.63±3.65^a^ | 7.70±3.32^ab^ | 3.40±1.14^b^ | Core | Fruity, Waxy |
| Cyclohexene, 1-methyl-4-(1-methylethylidene)- | 586-62-9 | 18.45 | 2.04 | 1040.00 | 0.25±0.03^a^ | 0.21±0.02^a^ | 0.55±0.77^a^ | 0.32±0.06^a^ | 0.33±0.21^a^ | Core | Woody, Citrus, Herbal |
| Bicyclo [2.2.1] hept-2-ene, 1,7,7-trimethyl- | 464-17-5 | 23.65 | 2.46 | 1177.14 | 0.36±0.05^a^ | 0.18±0.04^a^ | 0.39±0.18^a^ | 0.33±0.07^a^ | 0.33±0.23^a^ | Core | Camphoraceous, Pine |
| Benzoic acid, 2-hydroxy-, ethyl ester | 118-61-6 | 25.25 | 2.85 | 1212.12 | 0.03±0.00^a^ | 0.68±0.11^a^ | 0.52±0.74^a^ | 0.03±0.04^a^ | 0.54±0.76^a^ | Core | Wintergreen, Minty |
| Benzene, pentyl- | 538-68-1 | 20.98 | 2.22 | 1120.00 | 0.49±0.28^b^ | 0.26±0.14^b^ | 0.65±0.44^b^ | 2.16±0.19^a^ | 0.59±0.09^b^ | Core | Gasoline, olvent |
| 8-Methylnonanoic acid, ethyl ester | 54947-74-5 | 27.92 | 1.95 | 1272.74 | 0.89±1.26^a^ | 0.74±1.01^a^ | 5.49±4.25^a^ | 9.78±4.53^a^ | 1.64±1.07^a^ | Core | Waxy, Fruity |
| 3-Buten-2-one, 4-(2,6,6-trimethyl-1-cyclohexen-1-yl)- | 14901-07-6 | 32.05 | 2.73 | 1361.46 | 1.75±0.71^ab^ | 0.55±0.78^b^ | 0.00±0.00^b^ | 3.33±1.13^a^ | 0.54±0.23^b^ | Core | Violet, Berry, Woody |
| 2-Tridecanone | 593-08-8 | 32.18 | 2.07 | 1364.27 | - | 0.04±0.01^a^ | 0.16±0.09^a^ | 0.15±0.10^a^ | 0.04±0.05^a^ | Core | Waxy, Herbaceous, Mushroom |
| 2-Propenal, 3-(2,6,6-trimethyl-1-cyclohexen-1-yl)- | 4951-40-0 | 29.38 | 2.69 | 1305.59 | 0.09±0.01^a^ | 0.10±0.01^a^ | 0.21±0.09^a^ | 0.25±0.15^a^ | 0.06±0.09^a^ | Core | Saffron, Spicy, Hay |
| 2H-Pyran, 2-ethenyltetrahydro-2,6,6-trimethyl- | 7392-19-0 | 13.92 | 1.82 | 946.07 | 0.02±0.03^b^ | 0.05±0.02^ab^ | 0.16±0.08^a^ | 0.05±0.02^ab^ | 0.01±0.02^b^ | Core | Floral, Rose, Geranium |
| 2-Furanmethanol, 5-ethenyltetrahydro-α, α, 5-trimethyl-, cis- | 5989-33-3 | 17.92 | 2.24 | 1019.99 | 1.12±0.11^ab^ | 0.82±0.29^b^ | 1.88±0.71^a^ | 1.24±0.09^ab^ | 0.67±0.24^b^ | Core | Floral, Linden, Camphoraceous |
| 1-Hexanol, 2-ethyl- | 104-76-7 | 22.58 | 1.63 | 1154.28 | 1.25±0.93^b^ | 0.61±0.29^b^ | 0.98±0.13^b^ | 4.09±1.64^a^ | 1.31±0.05^b^ | Core | Fruity, Green, Herbaceous |
| 1-Decanol | 112-30-1 | 14.98 | 2.02 | 962.66 | 75.18±3.87^ab^ | 26.18±1.36^b^ | 91.08±1.25^ab^ | 134.22±3.98^a^ | 62.70±1.71^ab^ | Core | Waxy, Fatty, Citrus |
| 1,3,6-Octatriene, 3,7-dimethyl-, (Z)- | 3338-55-4 | 16.85 | 1.89 | 991.70 | 0.37±0.00^a^ | - | 0.20±0.28^a^ | 0.11±0.15^a^ | 0.35±0.50^a^ | Core | Citrus, Floral, Waxy |
| Undecanoic acid, ethyl ester | 627-90-7 | 31.12 | 1.91 | 1341.92 | - | 0.09±0.02^a^ | 0.30±0.39^a^ | - | 0.12±0.06^a^ | Differential | Waxy, Fruity, Coconut |
| Styrene | 100-42-5 | 10.72 | 2.38 | 893.34 | 0.11±0.03^ab^ | - | 0.20±0.10^a^ | 0.07±0.02^b^ | - | Differential | Gasoline, Plastic, Sweet |
| Pyrazine, 2-methoxy-3-(2-methylpropyl)- | 24683-00-9 | 21.92 | 2.48 | 1140.01 | 0.24±0.05^a^ | 0.13±0.03^ab^ | 0.29±0.10^a^ | - | 0.28±0.15^a^ | Both | Green Pepper, Earthy |
| Phenol, 2-methoxy- | 90-05-1 | 18.45 | 3.27 | 1040.02 | 1.07±0.06^a^ | 0.38±0.10^b^ | 0.26±0.09^ab^ | 0.44±0.17^b^ | 0.07±0.05^c^ | Differential | Smoky, Medicinal, Sweet |
| Octadecanoic acid, ethyl ester | 111-61-5 | 49.92 | 2.02 | 2057.16 | 1.32±0.87^a^ | 0.15±0.01^b^ | 0.74±0.21^ab^ | 0.57±0.27^ab^ | 0.24±0.06^ab^ | Differential | Waxy, Fatty |
| Naphthalene, 1-methyl- | 90-12-0 | 25.92 | 3.33 | 1227.29 | - | 0.14±0.01^a^ | - | - | 0.08±0.04^b^ | Differential | Tar, Mothball |
| Naphthalene, 1,2,3,4-tetrahydro-1,1,6-trimethyl- | 475-03-6 | 23.12 | 2.19 | 1165.73 | - | 0.02±0.00^a^ | - | - | 0.01±0.01^b^ | Differential | Woody, Camphoraceous |
| Naphthalene | 91-20-3 | 22.05 | 3.41 | 1142.86 | 0.14±0.04^a^ | - | - | - | 0.04±0.03^b^ | Differential | Mothball, Tar |
| Fenchol | 1632-73-1 | 24.05 | 2.55 | 1185.73 | - | - | 0.21±0.08^a^ | - | - | Differential | Camphoraceous, Pine |
| Ethyl 9-tetradecenoate | 50486-36-1 | 38.98 | 2.05 | 1484.91 | - | - | - | 0.64±0.22^a^ | 0.05±0.05^b^ | Differential | Waxy, Fruity |
| Decanoic acid, ethyl ester | 110-38-3 | 29.12 | 1.98 | 1300.01 | - | - | 1.94±0.93^a^ | - | - | Differential | Waxy, Fruity, Brandy |
| Caryophyllene | 87-44-5 | 30.05 | 2.16 | 1319.57 | - | 0.13±0.02^a^ | - | - | - | Differential | Woody, Spicy, Clove |
| Butyl benzoate | 136-60-7 | 28.45 | 2.90 | 1284.86 | - | - | - | 0.26±0.07^a^ | - | Differential | Balsamic, Fruity |
| Butanoic acid, ethyl ester | 105-54-4 | 7.52 | 1.95 | 804.48 | - | 0.08±0.02^b^ | 0.74±0.36^a^ | 0.32±0.08^ab^ | - | Differential | Fruity, Pineapple |
| Butanoic acid, 3-methyl-, ethyl ester | 108-64-5 | 9.25 | 1.97 | 852.62 | 0.43±0.30^a^ | 0.18±0.13^a^ | - | - | - | Differential | Fruity, Apple |
| Benzeneacetic acid, ethyl ester | 101-97-3 | 24.18 | 3.19 | 1188.58 | 0.36±0.02^b^ | 0.19±0.07^b^ | 1.96±0.63^a^ | 0.37±0.14^b^ | 0.19±0.08^b^ | Differential | Honey, Floral |
| Acetic acid, 2-phenylethyl ester | 103-45-7 | 24.58 | 3.18 | 1197.15 | - | 0.11±0.07^b^ | 0.68±0.20^a^ | 0.20±0.04^b^ | 0.26±0.11^b^ | Differential | Floral, Rose, Honey |
| 3-Cyclohexene-1-ethanol, β,4-dimethyl- | 18479-68-0 | 25.92 | 2.74 | 1227.29 | - | 0.17±0.01^b^ | - | 0.65±0.17^a^ | 0.16±0.10^b^ | Differential | Floral, Rose, Citrus |
| 2H-Pyran, 3,6-dihydro-4-methyl-2-(2-methyl-1-propenyl)- | 1786-08-9 | 21.52 | 2.40 | 1131.44 | - | 0.06±0.00^a^ | - | - | - | Differential | Herbal, Minty, Camphoraceous |
| 2-Heptanone | 110-43-0 | 10.72 | 2.33 | 893.36 | - | 0.03±0.00^a^ | - | - | - | Differential | Fruity, Cheese, Soapy |
| 2-Butanone, 4-(2,6,6-trimethyl-2-cyclohexen-1-yl)- | 31499-72-6 | 29.92 | 2.55 | 1316.77 | - | 0.04±0.00^b^ | - | 0.15±0.08^a^ | - | Differential | Violet, Berry, Woody |
| 2-Butanone, 4-(2,6,6-trimethyl-1-cyclohexen-1-yl)- | 17283-81-7 | 30.58 | 2.53 | 1330.74 | 0.44±0.12^ab^ | 0.38±0.16^b^ | 0.19±0.09^b^ | 0.78±0.19^a^ | 0.25±0.13^b^ | Both | Violet, Woody |
| 2,6-Octadien-1-ol, 3,7-dimethyl-, (Z)- | 106-25-2 | 24.45 | 2.44 | 1194.29 | - | - | 0.11±0.06^a^ | 0.13±0.03^a^ | 0.06±0.04^ab^ | Differential | Floral, Citrus, Waxy |
| 2(4H)-Benzofuranone, 5,6,7,7a-tetrahydro-4,4,7a-trimethyl-, (R)- | 17092-92-1 | 33.52 | 4.21 | 1392.18 | 0.68±0.38^a^ | 0.16±0.12^a^ | 0.29±0.21^a^ | 0.25±0.18^a^ | 0.13±0.08^a^ | Differential | Coconut, Woody |
| 1-Pentanol | 71-41-0 | 5.65 | 1.89 | - | 0.88±0.09^a^ | 0.27±0.01^a^ | 2.81±2.14^a^ | 1.63±0.50^a^ | 2.46±1.25^a^ | Differential | Fruity, Balsamic |
| 1-Oxaspiro [4.5] dec-6-ene, 2,6,10,10-tetramethyl- | 36431-72-8 | 26.18 | 2.19 | 1233.35 | 0.02±0.01^b^ | 0.08±0.02^a^ | - | - | - | Differential | Violet, Woody |
| 1-Cyclohexene-1-carboxaldehyde, 2,6,6-trimethyl- | 432-25-7 | 23.38 | 2.80 | 1171.43 | 0.10±0.03^ab^ | 0.16±0.09^a^ | 0.03±0.02^b^ | - | 0.10±0.05^ab^ | Differential | Saffron, Spicy |
| 1,6,10-Dodecatrien-3-ol, 3,7,11-trimethyl-, (E)- | 40716-66-3 | 34.18 | 2.24 | 1404.90 | 0.24±0.12^b^ | 0.22±0.11^b^ | - | 0.85±0.07^a^ | 0.15±0.04^bc^ | Differential | Woody, Floral |

**Table S3** Flavor substances in SCP (The metabolites, with variance importance for projection (VIP) value above 1.0 were considered to be potential biomarkers).

| Class | Name | m/z | rt(s) | Adduct | VIP |
| --- | --- | --- | --- | --- | --- |
| Amino acids | Serine | 104.04 | 369.64 | [M-H]- | 1.17 |
|  | Phenylalanine | 164.07 | 252.71 | [M-H]- | 2.84 |
|  | Ng, ng-dimethyl-l-arginine | 203.15 | 481.23 | [M+H]+ | 4.28 |
|  | L-pyroglutamic acid | 128.04 | 303.39 | [M-H]- | 13.16 |
|  | L-Glutamate | 192.02 | 391.97 | (M-H+2Na)+ | 1.15 |
|  | L-aspartic acid | 134.04 | 424.21 | [M+H]+ | 1.54 |
|  | L-Arginine | 175.12 | 527.27 | (M+H)+ | 3.98 |
|  | Glutamic acid | 146.05 | 391.77 | [M-H]- | 2.03 |
|  | DL-arginine | 175.12 | 498.83 | [M+H]+ | 8.58 |
|  | Betaine | 118.09 | 271.27 | [M+H]+ | 13.24 |
|  | Aspartic acid | 132.03 | 397.56 | [M-H]- | 5.31 |
| lipids | Thymol-beta-d-glucoside | 311.17 | 28.72 | [M-H]- | 5.64 |
|  | Sebacic acid | 203.12 | 115.69 | [M+H]+ | 1.24 |
|  | Palmitic acid alkyne | 253.22 | 122.37 | [M+H]+ | 2.09 |
|  | Oleamide | 282.28 | 35.54 | [M+H]+ | 15.15 |
|  | Mesaconic acid | 129.02 | 441.14 | (M-H)- | 1.83 |
|  | Lpc 18:2 | 520.34 | 188.55 | [M+H]+ | 1.25 |
|  | Linoleic acid | 279.23 | 46.76 | [M-H]- | 21.15 |
|  | Glycerophosphocholine | 104.11 | 220.29 | [M+H-C3H7O5P]+ | 6.97 |
|  | Erucamide | 338.34 | 35.42 | [M+H]+ | 3.91 |
|  | Eicosapentaenoic acid | 285.22 | 50.60 | (M+H-H2O)+ | 2.09 |
|  | Cis-4,7,10,13,16,19-docosahexaenoic acid | 329.25 | 34.53 | [M+H]+ | 2.07 |
|  | 9-hydroxy-5z,7e,11z,14z-eicosatetraenoic acid | 319.23 | 51.42 | [M-H]1- | 4.26 |
|  | 5-aminovaleric acid betaine | 160.13 | 381.29 | [M+H]+ | 1.16 |
|  | 2-arachidonoyl-1-stearoyl-sn-glycero-3-phosphoethanolamine | 766.52 | 138.34 | [M-H]- | 1.19 |
|  | 2-(5-oxovaleryl) phosphatidylcholine | 594.40 | 128.50 | [M+H]+ | 6.78 |
|  | 1-stearoyl-2-hydroxy-sn-glycero-3-phosphocholine | 524.37 | 173.50 | [M+H]+ | 1.13 |
|  | 1-palmitoyl-sn-glycero-3-phosphocholine | 496.34 | 189.97 | [M+H]+ | 1.59 |
|  | 1-palmitoyl-2-docosahexaenoyl-sn-glycero-3-phosphocholine | 806.55 | 39.25 | [M+H]+ | 1.45 |
|  | 1-palmitoyl-2-docosahexaenoyl-sn-glycero-3-phospho-(1'-rac-glycerol) | 793.52 | 39.23 | [M-H]- | 1.88 |
|  | 1-oleoyl-sn-glycero-3-phosphocholine | 522.35 | 187.51 | [M+H]+ | 2.68 |
|  | 1-hexadecanoyl-2-octadecadienoyl-sn-glycero-3-phosphocholine | 758.57 | 144.97 | [M+H]+ | 2.55 |
|  | 1-heptadecanoyl-sn-glycero-3-phosphocholine | 548.32 | 126.06 | [M+K]+ | 1.08 |
|  | 1,2-dipalmitoleoyl-sn-glycero-3-phosphocholine | 752.50 | 79.85 | [M+Na]+ | 1.70 |
|  | 1,2-dilinoleoyl-sn-glycero-3-phosphocholine | 782.57 | 143.07 | [M+H]+ | 3.04 |
| Organic acids | Taurine | 124.01 | 296.95 | [M-H]- | 1.37 |
|  | Pyruvate | 87.01 | 124.40 | [M-H]- | 1.29 |
|  | Malate | 133.01 | 402.88 | [M-H]- | 5.24 |
|  | L-carnosine | 156.08 | 397.53 | [M+H-C3H5ON]+ | 3.30 |
|  | Citrate | 191.02 | 464.79 | [M-H]- | 9.98 |
|  | Anserine | 241.15 | 372.22 | [M+H]+ | 1.05 |
| Peptides | Pro-Glu | 245.11 | 410.58 | [M+H]+ | 1.13 |
|  | L-glutathione, reduced | 288.08 | 69.80 | [M-H-H2O]- | 2.61 |
|  | Glu-Lys | 276.15 | 450.81 | [M+H]+ | 1.37 |
|  | Gamma-glutamylvaline | 247.18 | 269.96 | [M+H]+ | 3.39 |
|  | Gamma-glu-glu | 276.12 | 423.98 | [M+H]+ | 1.34 |
|  | Ala-Lys | 218.15 | 429.81 | [M+H]+ | 2.50 |

**Fig. S3.** Taste Sensory Evaluation Radar Chart of Five SCPs
